# A catalog of proteins released from *Asaia bogorensis* under two growth conditions

**DOI:** 10.1128/spectrum.01506-25

**Published:** 2025-08-29

**Authors:** Anna B. Manges, David J. Lampe

**Affiliations:** 1 Department of Biological Sciences, Duquesne University 6613, , Pittsburgh, Pennsylvania, USA; Brigham Young University, Provo, Utah, USA

**Keywords:** paratransgenesis, malaria, protein secretion, *Asaia bogorensis*

## Abstract

Bacterial secretion systems are essential for growth and responsible for a plethora of functions, ranging from nutrient acquisition to biofilm formation and pathogenesis. Bacteria use type II secretion systems (T2SSs) to transport folded proteins from the periplasm outside of the cell in a two-step process. Opportunistic pathogens often secrete virulence factors through this pathway. Type I secretion systems transport proteins across the inner and outer membranes in a single step. The type IV secretion system also transports substrates across the entire cell envelope. It may also translocate substrates directly into target cells. Researchers have utilized the gram-negative bacterium *Asaia bogorensis* for paratransgenesis. The bacterium was engineered to secrete anti-*Plasmodium* effector peptides to target the *Plasmodium* parasites that cause malaria in the mosquito vector through the T2SS. Here, we identify a catalog of proteins released by *A. bogorensis* under two growth conditions, as well as predictions for the secretion systems that transport them, using liquid chromatography with tandem mass spectrometry. We identified 343 proteins from both growth conditions, with 32 uniquely identified on chocolate agar and 82 proteins from minimal media only. The substrates secreted by the systems identified here may be utilized in the future to improve paratransgenesis. We propose utilizing the type I and/or type IV secretion systems for more streamlined effector secretion directly out of the bacterium, which may improve strain fitness. Fusion of anti-parasitic effector peptides to the substrates encoded at these loci could also serve as an effective minimal paratransgenic strain not reliant upon plasmids.

**IMPORTANCE:**

Bacterial secretion systems are responsible for nutrient acquisition, biofilm formation, and pathogenesis. *Asaia bogorensis* is a gram-negative bacterium used for paratransgenesis. The bacterium was engineered to secrete anti-*Plasmodium* effectors to prevent development of the *Plasmodium* parasites that cause malaria in the mosquito vector. Here, we identify a catalog of proteins released by *A. bogorensis* under two growth conditions as well as predictions for the secretion systems that transport them. These secretion systems may be used in the future to improve paratransgenesis. We propose utilizing single-step secretion systems for more streamlined effector secretion from the bacterium. Fusion of anti-parasitic effector peptides to the substrates encoded at these loci could serve as an effective minimal paratransgenic strain not reliant upon plasmids. Improvement of secretion by paratransgenic bacteria would allow for more effective interference with *Plasmodium* parasites in their mosquito vectors. More effective paratransgenesis has the potential to prevent cases of malaria worldwide.

## INTRODUCTION

Bacterial secretion systems are responsible for transporting proteins from the cytoplasm to the cell membrane, extracellular environment, and even to other bacteria or eukaryotes. The proteins exported by secretion systems have many functions, ranging from nutrient acquisition and adhesion to involvement in pathogenesis and competition with other microorganisms, and even transport of DNA molecules (1, 2). Bacterial secretion is essential for growth, with some secretion systems conserved in a wide range of bacteria and others much more specific; these may be found in only some bacterial species or be responsible for secretion of only a small number of proteins for a specific purpose (3).

The general secretory (Sec) pathway and the twin arginine translocation (Tat) pathway are the most highly conserved secretion mechanisms across life and are responsible for the majority of protein transport across the cytoplasmic membrane (4). From here, gram-negative bacteria either utilize another secretion system to transport proteins out of the cell, or they remain in the periplasm. They may also be integrated into the cell membrane. Other secretion systems do not rely upon the Sec or Tat pathways and instead are responsible for secretion across both bacterial membranes. These secretion systems are Sec- and Tat-independent and form channels that span both the inner and outer membranes.

Type II secretion systems (T2SSs) are used by bacteria to transport folded proteins from the periplasm to the extracellular environment and are often key virulence factors for human opportunistic pathogens. The T2SS is a two-step process. Proteins marked for secretion are carried across the inner membrane and into the periplasm of gram-negative bacteria by either the Tat or Sec systems. The proteins finish folding here, followed by transportation across the outer membrane upon completion via the dedicated T2SS machinery (5). The type I secretion system (T1SS), in contrast, is simpler. T1SSs are Sec-independent and transport their substrates in an unfolded state out of the cell in a one-step process. Found in a wide range of gram-negative bacteria, T1SS substrates include enzymes, such as proteases and lipases, as well as heme-binding proteins and adhesins (6).

Type IV secretion systems (T4SSs) are found in gram-negative bacteria, gram-positive bacteria, and archaea (7). These secretion systems are highly complex and have two primary functions: DNA transfer (conjugation) and direct protein substrate transfer, primarily through direct donor-target cell contact (8, 9). Many gram-negative species rely upon T4SSs for pathogenicity, delivering effector molecules to other bacteria and, in some cases, to fungal, plant, and mammalian cells (8, 10). Gram-negative bacterial pathogens employ T4SSs to transfer virulence factors to host cells during infection. T4SSs span the gram-negative cell envelope and contain periplasm-spanning channels that enable secretion of proteins directly from the cytoplasm to the extracellular environment or into target cells (3).

Proteins are directed for secretion out of the cell by secretion signals. Secretion signals are short peptides, generally ranging from 16 to 30 amino acids present at the end of a newly synthesized protein that directs transport of the protein out of the cell (3). Secretion signals can be located on either the N-terminus or C-terminus of proteins and may be cleaved from the protein. The Sec and Tat pathways recognize N-terminal signal sequences to direct proteins to the periplasm/inner membrane. Proteins with N-terminal signal sequences are also directed to the T2SS for secretion out of the cell. Proteins secreted by T1SSs and T4SSs, in contrast, have signal sequences found within the C-terminal 100 amino acids (11).

Biologists rely on efficient bacterial secretion across numerous disciplines, including vaccine therapeutics, industrial applications such as biofuel production and bioremediation, and prevention of vector-borne diseases (12, 13). One promising technique being developed for controlling vector-borne diseases is paratransgenesis (14). Paratransgenesis is the engineering of symbiont microorganisms to produce antipathogen effector molecules inside of a vector organism, and it relies on one key aspect: secretion. If the effector is not efficiently secreted, then it cannot optimally target and kill the parasite in the extracellular milieu. Researchers using paratransgenesis for malaria prevention have successfully engineered *Asaia bogorensis*, a gram-negative bacterium, that releases the antimicrobial peptide scorpine and prevents *Plasmodium berghei* parasite development. Grogan et al. modified the antimicrobial peptide scorpine with 20 native Sec or Tat signal sequences predicted to be associated with the T2SS. They hypothesized these signal sequences would improve protein direction out of the cell (15). Several of these signals significantly improved extracellular levels of scorpine and suppressed *P. berghei* in *Anopheles stephensi* mosquitoes. But the reduction was not absolute, and the fitness of the paratransgenic bacteria was significantly worse than that of the wild-type bacterium. Thus, secretion of the anti-parasitic effectors might be improved not only to increase the fitness of the bacteria but also to optimize anti-*Plasmodium* effects.

Recently, Wang et al. developed BastionHub, a universal platform for integrating and analyzing multiple types of secreted proteins by gram-negative bacteria (16). BastionHub utilizes available data from the five major secretion systems and machine learning-based prediction tools. It can be used to identify and predict the secretion of proteins based on a variety of structural components (16). Analysis of the *Asaia* genome using this software predicts close to 800 potentially secreted products, a number much too large to test experimentally. Furthermore, there is no experimental evidence in the literature identifying any native *Asaia* secretions. As such, identifying molecules that are released by *A. bogorensis* under normal growth conditions and under conditions mimicking those in the mosquito midgut environment when contact with *Plasmodium* occurs is critical. This project focused on collecting and cataloging proteins released by *A. bogorensis* under two conditions and identifying these using liquid chromatography with tandem mass spectrometry (LC-MS/MS). *A. bogorensis* was originally identified in plant nectar and is a frequent component of the mosquito microbiome (17–19). The strain used in this study was isolated from a colony of *Anopheles stephensi* mosquitoes (18). As such, we chose two growth media to mimic these conditions. The first, a minimal media, was chosen as a baseline for growth in the environment or in a sugar source. It represents the growth of the bacterium in natural conditions outside of the mosquito. The second, a lysed red blood cell agar (chocolate agar), was selected to mimic the mosquito midgut environment during and immediately following a mosquito blood meal. This medium represents the conditions during which the bacterium would encounter *Plasmodium* parasites and is useful for identifying genes expressed only under these conditions. We provide here a catalog of proteins secreted by the bacterium and predicted modes of secretion using the program BastionX. Three hundred and forty-three proteins were identified from *Asaia* cultures grown on minimal agar medium and chocolate (lysed red blood cell) agar media. Thirty-two proteins were unique to the chocolate agar condition, with 22 of these predicted to be secreted.

## MATERIALS AND METHODS

### Collection of secreted samples

*Asaia bogorensis* SF2.1 was grown in mannitol broth (0.5% yeast extract, 0.3% peptone, 2.5% mannitol [wt/vol]) to saturation in triplicate. At this time, 100 µL of each culture was placed onto Davis minimal agar (0.7% dipotassium phosphate, 0.2% monopotassium phosphate, 0.05% sodium citrate, 0.01% magnesium sulfate, 0.1% ammonium sulfate [wt/vol] with 15 g/L agar) supplemented with 10% glucose solution (vol/vol), as well as chocolate agar (Davis minimal agar supplemented with 10% glucose solution [vol/vol] and 2.5% lysed defibrinated blood [vol/vol]). After 3 days of growth at 30°C, all samples and secretions were gently scraped from the plates for 30 seconds in 1 mL of Davis minimal broth using a glass scraper and immediately transferred to microcentrifuge tubes. Approximately 4.3 × 10^10^ cells per biological replicate were collected. All samples were centrifuged at 9,000 × *g* for 5 minutes, and supernatants were transferred immediately to fresh tubes and saved as the secreted samples. Three biological replicates were used for each growth condition.

### Sample preparation

Seventy-five microliters of each supernatant was moved to new microcentrifuge tubes with 25 µL of 3× Laemmli buffer and boiled for 8 minutes. Samples were allowed to cool and then placed at −80°C. A Bradford assay using the Pierce Micro BCA Protein Assay Kit (Product #23235) was then performed according to the manufacturer’s instructions to quantify the concentrations of proteins in all samples against a Pierce Albumin Standard (Product #23209). For the assay, 1.5 µL of each sample was added to 148.5 µL of Davis minimal broth and 150 µL of the Bradford working reagent in a 96-well plate, using two replicates of each standard and sample. The absorbance was measured using the SpectraMax ID3 plate reader at 562 nm. The protein concentration of each sample was determined against the standard curve. As specified in the kit, the BSA standards ranged from 0 to 250 µg/mL. Three hundred fifty micrograms of protein for each sample was loaded onto a 1D SDS gel and run for 5 minutes at 200V until samples were 2 mm–3 mm below the bottom of each well. The gel was stained with Coomassie, and protein bands were excised from the gel and stored in 100 µL of 5% acetic acid, followed by immediate shipment to the Michigan State University Proteomics Core Facility for liquid chromatography with tandem mass spectrometry analysis.

### LC-MS/MS and data analysis

The Michigan State University Proteomics Core Facility performed protein sample extraction from the 1D SDS gel, sample preparation, and LC-MS/MS. Charge state deconvolution and deisotoping were not performed. All MS/MS samples were analyzed using Mascot (Matrix Science, London, UK; version 2.8.3). The Mascot search engine was set up to search the crap_20230111.fasta, UP_human_20220418.fasta, and UP_A_Borogensis_20231003 databases (unknown version, 81,934 entries), assuming strict trypsin digestion. Mascot was searched with a fragment ion mass tolerance of 0.020 Da and a parent ion tolerance of 10.0 ppm. Carbamidomethylation of cysteine was specified in Mascot as a fixed modification. Oxidation of methionine was specified in Mascot as a variable modification.

Scaffold (version Scaffold_5.3.0, Proteome Software Inc., Portland, OR) was used to validate MS/MS-based peptide and protein identifications. Peptide identifications were accepted if they could be established at greater than 95.0% probability by the Percolator posterior error probability calculation (20). Protein identifications were accepted if they could be established at greater than 84.0% probability to achieve an false discovery rate (FDR) of less than 1.0% and contained at least one identified peptide. Protein probabilities were assigned by the Protein Prophet algorithm (21). Proteins that contained similar peptides and could not be differentiated based on MS/MS analysis alone were grouped to satisfy the principles of parsimony. Proteins sharing significant peptide evidence were grouped into clusters. This portion of the data analysis was performed by the Michigan State University Proteomics Core Facility.

All proteins identified as *Homo sapiens* were excluded from analysis, as these were from the red blood cells present in the chocolate agar. Any proteins only found in one biological replicate for each growth condition were also excluded from analysis. Proteins identified from both minimal and chocolate agar conditions were included if found in one biological replicate from each condition group. A list of all proteins identified from a single sample is located in Table S7. Proteins found in each experimental group were recorded and entered into the BastionX modeling software to predict the probability of secretion and which secretion system was most likely responsible. Version 1.0 of the prediction software was used, predicting for all secretion systems and accurate mode selected. As per the program pipeline, proteins with a probability greater than 50% were predicted to be secreted. All possible secretion system predictions are detailed in the supplemental information, with only the most probable secretion systems described in Tables 1 to 4.

**TABLE 1:** Proteins identified on chocolate media agar and prediction of secretion^*a*^^,^^*b*^

Protein name	Gene name	Accession	Predicted secretion system	BastionHub prediction results
DUF3309 domain-containing protein		A0A433WW75	I	0.807
Carboxypeptidase-related protein		A0A060QFN3	II	1
TonB-dependent receptor		A0A060QDK6	II	0.762
Acid phosphatase	phoC	A0AAN4U1B7	II	0.681
Peptidase		A0AAN4R7N1	II	0.973
Outer membrane lipoprotein omlA		A0AAN4R4A8	II	0.535
Carbohydrate-selective porin		A0AAN4R463	II	0.956
Carbohydrate-selective porin		A0A060QGH0	II	1
Peptidase S10		A0AAN4R155	II	1
DUF2272 domain-containing protein		A0A060QD52	II, VI	45658
Porin		A0A060QIF4	II	1
Peptidase C51 domain-containing protein		A0A060QLL6	II	0.831
Cell wall-associated hydrolase		A0AAN4R1B4	II, VI	1, 1
Putative lipoprotein		A0A060QKU7	II	0.81
Uncharacterized protein		A0AAN4R1Y5	II	0.653
Lipoprotein		A0A060QD84	II	0.86
Lipoprotein		A0A060QI71	III	1
Transcriptional initiation protein Tat		A0A060QG54	III	0.999
Lipoprotein		A0A060QI19	III	0.513
Uncharacterized protein		A0AAN4U2I1	III	0.984
YkuD domain-containing protein		A0A060QHE7	III	0.955
Lipoprotein		A0A0P0YGH5	III	0.718
DUF333 domain-containing protein		A0AAN4R188	N/A	N/A
Lytic transglycosylase		A0AAN4R3Q4	No	N/A
Putative dihydroxyacetone kinase, dihydroxyacetone-binding subunit		A0A060QLZ6	No	N/A
Probable periplasmic serine endoprotease DegP-like		A0A060QHI0	No	N/A
Alkaline phosphatase family protein		A0AAN4R3Z5	No	N/A
Putative cytochrome c-552		A0A060QBQ6	No	N/A
Biopolymer transport protein ExbB		A0A060QGG7	No	N/A
Lipopolysaccharide assembly protein A domain-containing protein		A0A060QJW1	No	N/A
Aspartate aminotransferase family protein		A0AAN4U1J6	No	N/A
D-amino acid oxidase		A0A060QKK3	No	N/A

^*a*^ N/A: no BastionHub prediction for secretion.

^*b*^ Gene name cells are empty if there is no available annotated gene name.

**TABLE 2:** Proteins identified on minimal media agar and prediction of secretion^*a*^^,^^*b*^

Protein name	Gene name	Accession	Predicted secretion system	BastionHub prediction results
Uncharacterized protein		A0AAN4R2X5	I	0.756
Bulb-type lectin domain-containing protein		A0AAN4U1B4	I	0.972
Basal-body rod modification protein FlgD		A0A060QIM2	II	0.985
Flagellar basal-body rod protein FlgG		A0AAN4R026	II	0.829
Sucrose isomerase/alpha amylase		A0A0P0YFZ2	II	0.722
SPOR domain-containing protein		A0AAN4R683	II	0.601
Gluconolactonase		A0A060QJ35	II	0.83
Flagellar hook-basal body complex protein FliE	fliE	A0AAN4R3E9	III	0.7
DUF3597 domain-containing protein		A0A060QIH8	III	0.795
GcrA cell cycle regulator domain protein		A0A060QHS5	III	0.769
Uncharacterized protein		A0AAN4R2J3	III	0.582
Flagellar basal body rod protein FlgB		A0A060QIL6	III	0.838
Pentapeptide MXKDX repeat protein		A0A060QKH2	III	1
Exodeoxyribonuclease seven small subunit	xseB	A0A060QFS4	III	0.575
DUF4167 domain-containing protein		A0A0P0YFQ5	III	0.995
Cytoplasmic protein		A0A060QI69	IV	0.919
Phosphoketolase		A0AAN4U2Z8	VI	0.835
Peptide methionine sulfoxide reductase MsrB	msrB	A0A060QFX0	VI	0.63
Cupin domain protein		A0A0P0YDP2	VI	1
50S ribosomal protein L31	rpmE	A0AAN4U452	No	N/A
Electron transfer flavoprotein subunit beta	etfB	A0AAN4U493	No	N/A
Uncharacterized protein		A0AAN4R4B5	No	N/A
Peptidase C56 PfpI		A0AAN4R1Z1	No	N/A
Oxidoreductase		A0AAN4R344	No	N/A
Trehalose 6-phosphate phosphatase	ostB	A0AAN4U3N5	No	N/A
Acetylornithine aminotransferase		A0A0P0YJ19	No	N/A
Xanthine-guanine phosphoribosyltransferase	gpt	A0AAN4U365	No	N/A
Adenylate kinase	adk	A0AAN4R1Q5	No	N/A
Dihydroxy-acid dehydratase	ilvD	A0AAN4R3I2	No	N/A
Phosphoribosylformylglycinamidine cyclo-ligase	purM	A0AAN4R0X8	No	N/A
Thiol:disulfide oxidoreductase	yfcG	A0AAN4U3S2	No	N/A
Uncharacterized protein		A0AAN4U262	No	N/A
Cell division protein FtsZ	ftsZ	A0AAN4U3Y8	No	N/A
Enolase-phosphatase E1	mtnC	A0AAN4R5T7	No	N/A
Phosphoglycerate mutase		A0AAN4U3H2	No	N/A
S-methyl-5'-thioadenosine phosphorylase	mtnP	A0AAN4R0K0	No	N/A
dTDP-4-dehydrorhamnose 3,5-epimerase		A0AAN4R541	No	N/A
Haloacid dehalogenase		A0AAN4U2W3	No	N/A
Methylthioribulose-1-phosphate dehydratase	mtnB	A0AAN4R296	No	N/A
Uncharacterized protein		A0AAN4R1W2	No	N/A
ATPase		A0AAN4R216	No	N/A
Uncharacterized protein		A0AAN4U2H7	No	N/A
Xylulose kinase	xylB	A0AAN4R5K4	No	N/A
Glycosyl hydrolase		A0AAN4R638	No	N/A
3'(2'),5'-Bisphosphate nucleotidase CysQ	cysQ	A0AAN4U2M7	No	N/A
Ribulose-phosphate 3-epimerase	rpe	A0AAN4U269	No	N/A
Glutamate-cysteine ligase	gsh1	A0AAN4R407	No	N/A
Phosphoserine aminotransferase		A0AAN4R1N4	No	N/A
Integration host factor subunit beta	ihfB	A0A060QEZ9	No	N/A
3-Oxoacyl-[acyl-carrier-protein] synthase 2	fabF	A0AAN4U2R1	No	N/A
GAF domain-containing protein		A0A060QJ84	No	N/A
Glutaredoxin		A0AAN4R7C1	No	N/A
RNA polymerase-binding transcription factor DksA	dksA	A0AAN4U273	No	N/A
Molybdenum cofactor biosynthesis protein B	moaB	A0AAN4R408	No	N/A
Peptidase T4		A0AAN4R010	No	N/A
Peptidyl-prolyl cis-trans isomerase		A0AAN4R5V7	No	N/A
FMN-dependent NADH-azoreductase	acpD	A0AAN4R5X4	No	N/A
Adenine phosphoribosyltransferase	apt	A0AAN4U255	No	N/A
Thioredoxin-dependent peroxiredoxin		A0A060QI59	No	N/A
NAD(P)-dependent oxidoreductase		A0AAN4R1S4	No	N/A
Phosphatase		A0AAN4U2D8	No	N/A
Histidinol-phosphatase		A0AAN4R719	No	N/A
Uroporphyrinogen decarboxylase	hemE	A0A0P0YK53	No	N/A
Farnesyltranstransferase		A0AAN4R4W3	No	N/A
Uncharacterized protein		A0AAN4R4V8	No	N/A
Glucokinase		A0AAN4U1Z0	No	N/A
50S ribosomal protein L20	rplT	A0AAN4R5H6	No	N/A
Site-determining protein	minD	A0AAN4R2Z7	No	N/A
Aldose 1-epimerase		A0AAN4U2E0	No	N/A
Uracil phosphoribosyltransferase	upp	A0AAN4R645	No	N/A
Ferredoxin-NADP reductase		A0AAN4U235	No	N/A
Phosphoglycolate phosphatase	pgp	A0AAN4R2H9	No	N/A
3-Oxoacyl-ACP reductase		A0AAN4R3D0	No	N/A
Ferric uptake regulation protein	fur	A0A060QKA4	No	N/A
Inositol-1-monophosphatase		A0A060QE01	No	N/A
Thioredoxin reductase		A0AAN4R4F8	No	N/A
NAD(P)-binding domain-containing protein		A0A060QJ50	No	N/A
Aldo/keto reductase		A0AAN4R1X0	No	N/A
Methionine aminopeptidase	map	A0A060QID3	No	N/A
Glutathione-disulfide reductase	gor	A0AAN4U2D5	No	N/A
Beta-ketoacyl-ACP reductase		A0AAN4R2L2	No	N/A
Anaphase-promoting complex subunit 4-like WD40 domain-containing protein		A0A060QJ87	No	N/A

^*a*^ N/A: no BastionHub prediction for secretion.

^*b*^ Gene name cells are empty if there is no available annotated gene name.

**TABLE 3:** All proteins identified under both growth conditions^*a*^^,^^*b*^

Protein name	Gene name	Accession	Predicted secretion system	BastionHub prediction results
Outer membrane protein		A0AAN4U3I6	I	1
Flagellar hook protein FlgE	flgE	A0AAN4R3W6	I, II	45292
Lipoprotein		A0AAN4U3B7	I	0.924
Outer membrane protein		A0AAN4R2L9	I	1
Lipoprotein		A0A060QD82	I	0.997
TonB-dependent outer membrane siderophore receptor		A0AAN4U233	II	0.944
TonB-dependent receptor		A0AAN4R251	II	0.904
TonB-dependent outer membrane colicin I receptor		A0A0P0YI97	II	0.918
YncE family protein		A0A0P0YE35	II	0.805
Flagellin		A0AAN4R5R2	II	0.569
Flagellar hook-associated protein 1		A0AAN4QZU3	II	0.718
Flagellar hook protein FlgE D2 domain-containing protein		A0AAN4R0D8	II	0.933
TonB-dependent receptor	btuB	A0AAN4R6T1	II	0.874
TonB-dependent outer membrane siderophore receptor		A0A0N7KVB0	II	0.904
TonB-dependent receptor		A0AAN4R0V8	II	0.944
TonB-dependent receptor		A0A0P0YHI2	II	1
TonB-dependent receptor-like beta-barrel domain-containing protein		A0AAN4R2F9	II	0.918
Lipoprotein		A0AAN4R288	II	0.896
Aldose 1-epimerase		A0A0P0YEH3	II	0.825
Peptidase M61		A0AAN4R1M7	II	0.99
FAS1 domain-containing protein		A0AAN4R766	II	0.792
TonB-dependent receptor		A0A0P0YEY9	II	0.944
Protein TolB	tolB	A0AAN4R0H1	II	0.954
Lipoprotein		A0A060QEU8	II	0.736
Lipase		A0AAN4R2H1	II	0.992
Ysc84 actin-binding domain-containing protein		A0AAN4R2R6	II	0.624
TonB-dependent siderophore receptor		A0A0P0YG72	II	0.848
membrane protein		A0AAN4R312	II	0.978
Outer membrane protein assembly factor BamB	bamB	A0AAN4R100	II	0.749
Glucans biosynthesis protein G		A0A060QIL1	II	0.573
Peptidoglycan lytic exotransglycosylase		A0AAN4U1L7	II	0.654
Probable outer membrane protein		A0A060QKA2	II	0.92
Leucine-binding protein domain-containing protein		A0AAN4R4V6	II	0.738
Phosphate-binding protein PstS	pstS	A0AAN4R2W7	II	0.971
Outer membrane protein OmpW		A0AAN4R498	II	0.86
Gluconolaconase	xylC	A0AAN4R0F1	II	0.56
Porin		A0AAN4R2I7	II	1
Ribonuclease I		A0AAN4R1H4	II	0.856
3-Carboxymuconate cyclase		A0AAN4R2Y8	II	0.971
Uncharacterized protein		A0AAN4R2S7	II	0.548
Peptidase		A0AAN4U369	II	0.65
Lytic murein transglycosylase B		A0A0P0YG35	II	0.886
Uncharacterized protein		A0AAN4R021	II	0.632
TonB-dependent outer membrane siderophore receptor		A0AAN4R0C6	II	0.944
C-type lysozyme inhibitor domain-containing protein		A0AAN4R1U7	II	0.753
Glycogen debranching enzyme		A0A060QIY2	II	0.974
Porin		A0AAN4U3E7	II	1
Endonuclease		A0AAN4R537	II	0.71
Maltose ABC transporter substrate-binding protein		A0AAN4R3M9	II	0.58
Acid phosphatase		A0AAN4R1R1	II	0.811
Ferric siderophore receptor	bfrH	A0AAN4R5L8	II	0.776
Thioredoxin domain-containing protein		A0AAN4R1W8	II	0.644
Pyrroloquinoline quinone predicted-dependent dehydrogenase		A0A0N7KVD1	II	1
Polyisoprenoid-binding protein		A0AAN4R2U9	II	0.526
Outer membrane protein assembly factor BamA	bamA	A0A060QGH4	II	0.555
Glutaryl-7-ACA acylase	gaa	A0AAN4U2U5	II	0.972
Levanase		A0AAN4R1X4	II	1
Iron transport outer membrane receptor		A0AAN4R4Z7	II	0.817
Alpha,alpha-trehalase		A0AAN4U3F6	II	0.96
Gluconolactonase		A0AAN4R0S4	II	0.821
Uncharacterized protein		A0AAN4R4K7	III	0.636
Uncharacterized protein		A0AAN4R0W4	III	0.976
Uncharacterized protein		A0A060QCJ9	III	0.967
Glycine zipper domain-containing protein		A0AAN4U2F3	III	0.9
DUF2125 domain-containing protein		A0AAN4R464	III	1
Uncharacterized protein		A0AAN4U3A6	III	0.826
Uncharacterized protein		A0A060QCG9	III	1
Secreted protein		A0AAN4R0G7	III	0.999
DUF2501 domain-containing protein		A0A060QJA9	III	1
Cell envelope biogenesis protein OmpA		A0A060QKK6	III	0.991
MucR family transcriptional regulator		A0AAN4R381	III	0.773
Proteinase		A0AAN4R1W1	III	0.538
Entericidin		A0AAN4U355	III	0.991
Uncharacterized protein		A0AAN4U2V8	III	0.664
Outer membrane protein OmpH/Skp		A0AAN4U1G0	III	0.595
Uncharacterized protein		A0A060QM33	III	1
Ankyrin repeat protein		A0AAN4R330	III	0.973
Large ribosomal subunit protein uL29	rpmC	A0A060QG13	III	0.684
DSBA oxidoreductase		A0AAN4R2A7	III	0.554
YfdX protein		A0AAN4R3K1	III	1
Periplasmic heavy metal sensor		A0AAN4R4L4	III	0.58
Uncharacterized protein		A0A060QDW4	III	0.975
Thiamine biosynthesis protein ThiC		A0A0P0YGH6	III	0.587
Lipoprotein		A0AAN4R2Z3	III	0.535
Integral membrane protein CcmA involved in cell shape determination		A0A060QLD3	III	0.51
Uncharacterized protein		A0A060QGF0	III	0.948
DUF3126 domain-containing protein		A0A060QIP7	III	0.506
DUF883 domain-containing protein		A0A060QK08	III	0.983
DUF2934 domain-containing protein		A0A060QAY9	III	0.959
Hypervirulence associated protein TUDOR domain-containing protein		A0A060QID8	IV, VI	0.632, 0.632
UrcA family protein		A0AAN4R0P5	IV	0.716
DUF1134 domain-containing protein		A0AAN4R5B0	VI	0.677
Large ribosomal subunit protein bL27	rpmA	A0A060QDR5	VI	0.621
LysM/phospholipid-binding domain protein		A0A060QHZ6	VI	0.556
Methionine adenosyltransferase		A0A433X0F8	VI	0.548
Lipoprotein		A0AAN4R2L1	VI	0.962
Uncharacterized protein		A0AAN4R0E4	VI	0.567
Hypothetical protein		A0A0P0YFS9	VI	0.938
Alginate biosynthesis protein AlgF		A0AAN4U3L3	VI	0.85
DUF3465 domain-containing protein		A0AAN4R038	VI	1
Gluconolaconase		A0AAN4R572	VI	0.855
Lipoprotein		A0AAN4R1S0	VI	0.85
SH3 domain-containing protein		A0A0P0YJB3	VI	1
Uncharacterized protein		A0AAN4R5K2	VI	0.865
Catalase	katA	A0AAN4U282	VI	0.828
Uncharacterized protein		A0AAN4R2A1	VI	0.803
Parvulin-like PPIase		A0A060QC01	No	N/A
Uncharacterized protein		A0AAN4R1Q0	No	N/A
Dipeptidyl carboxypeptidase II	dcp	A0AAN4U291	No	N/A
Uncharacterized protein		A0AAN4U190	No	N/A
Elongation factor Tu	tuf	A0A060QG23	No	N/A
Glyceraldehyde-3-phosphate dehydrogenase		A0A060QJP8	No	N/A
Uncharacterized protein		A0AAN4R4B3	No	N/A
Aconitate hydratase		A0AAN4R161	No	N/A
Uncharacterized protein		A0AAN4R3X0	No	N/A
Chaperonin GroEL	groEL	A0A060QBJ4	No	N/A
Small ribosomal subunit protein uS19	rpsS	A0A433WZ75	No	N/A
Transketolase		A0A060QI11	No	N/A
N-ethylmaleimide reductase		A0A060QJK2	No	N/A
Peptidoglycan-associated lipoprotein	pal	A0A060QBC2	No	N/A
Aldehyde dehydrogenase		A0AAN4R3I6	No	N/A
Large ribosomal subunit protein uL11	rplK	A0A060QHD3	No	N/A
Uncharacterized protein		A0AAN4R2T0	No	N/A
Zinc metalloprotease		A0AAN4R6B2	No	N/A
D-3-phosphoglycerate dehydrogenase		A0AAN4R4B4	No	N/A
Uncharacterized protein		A0AAN4R413	No	N/A
Alkyl hydroperoxide reductase C		A0A060QKS4	No	N/A
Non-specific DNA-binding protein Dps / Iron-binding ferritin-like antioxidant protein / Ferroxidase		A0A060QKS1	No	N/A
Isocitrate dehydrogenase		A0AAN4U3I3	No	N/A
6-Phosphogluconate dehydrogenase,decarboxylating		A0A060QBX4	No	N/A
Gamma-glutamyltranspeptidase		A0A0P0YI95	No	N/A
Small ribosomal subunit protein uS12	rpsL	A0A060QKQ0	No	N/A
Large ribosomal subunit protein bL35	rpmI	A0A060QGV0	No	N/A
Endopeptidase DegP/Do		A0A0P0YCQ8	No	N/A
Uncharacterized protein		A0AAN4R2G3	No	N/A
Aminotransferase	hisC	A0AAN4R5F7	No	N/A
Uncharacterized protein		A0AAN4R0X0	No	N/A
Large ribosomal subunit protein bL28	rpmB	A0A060QF16	No	N/A
Arginine biosynthesis bifunctional protein ArgJ	argJ	A0AAN4U1Q8	No	N/A
Osmotically inducible protein C		A0A060QHA3	No	N/A
Chaperone protein DnaK	dnaK	A0AAN4R7N6	No	N/A
Endoribonuclease L-PSP		A0A060QJF4	No	N/A
Glucose-6-phosphate 1-dehydrogenase	zwf	A0AAN4U1I0	No	N/A
Fructose-bisphosphate aldolase		A0AAN4R015	No	N/A
OmpA-like domain-containing protein		A0A060QJL6	No	N/A
Small ribosomal subunit protein bS20	rpsT	A0A060QKE8	No	N/A
Mannitol dehydrogenase	mtlD	A0AAN4U3M1	No	N/A
NAD-dependent malic enzyme		A0A0P0YI47	No	N/A
Membrane integrity-associated transporter subunit PqiC		A0A0P0YDS7	No	N/A
Large ribosomal subunit protein bL19	rplS	A0A060QEQ5	No	N/A
Superoxide dismutase		A0AAN4R0J4	No	N/A
Elongation factor Ts	tsf	A0A060QK90	No	N/A
S-(hydroxymethyl)glutathione dehydrogenase		A0AAN4U1Y7	No	N/A
Small ribosomal subunit protein uS17	rpsQ	A0A060QHB2	No	N/A
TPP-and FAD-dependent putative pyruvate dehydrogenase subunit A, peripheral membrane pyruvate oxidase poxB-like		A0A0P0YI64	No	N/A
Cold shock protein CspC		A0A060QJB1	No	N/A
UTP–glucose-1-phosphate uridylyltransferase		A0AAN4U2V9	No	N/A
Enolase	eno	A0AAN4R458	No	N/A
Large ribosomal subunit protein uL14	rplN	A0A060QL79	No	N/A
Uncharacterized protein		A0AAN4U1E6	No	N/A
Phosphoglucomutase, alpha-D-glucose phosphate-specific	celB	A0AAN4R0X4	No	N/A
Large ribosomal subunit protein bL17	rplQ	A0A433WZH2	No	N/A
Pyruvate kinase	pykF	A0AAN4R5E6	No	N/A
Large ribosomal subunit protein bL33	rpmG	A0A060QEQ7	No	N/A
Peroxiredoxin		A0AAN4U159	No	N/A
Alcohol dehydrogenase		A0A060QEJ3	No	N/A
Fructose-1,6-bisphosphatase		A0A060QK82	No	N/A
Outer membrane protein assembly factor BamD	bamD	A0AAN4U381	No	N/A
Large ribosomal subunit protein uL22	rplV	A0A060QL81	No	N/A
Glutamine synthetase		A0A060QLB9	No	N/A
50S ribosomal protein L18	rplR	A0AAN4U2L5	No	N/A
Uncharacterized protein		A0AAN4R1S2	No	N/A
Outer-membrane lipoprotein carrier protein		A0A060QDZ8	No	N/A
Phosphoglycerate kinase	pgk	A0AAN4R3L9	No	N/A
Transcriptional regulator, MarR family		A0A060QBI1	No	N/A
Nucleoside diphosphate kinase	ndk	A0A060QBH3	No	N/A
Uncharacterized protein		A0AAN4U1Y2	No	N/A
Mannitol dehydrogenase	mtlD	A0AAN4R2Z0	No	N/A
6-Phosphogluconolactonase		A0AAN4U1E2	No	N/A
Uncharacterized protein		A0AAN4U1T5	No	N/A
Small ribosomal subunit protein bS16	rpsP	A0A060QE38	No	N/A
Fumarate hydratase class II 2	fumC2	A0AAN4R470	No	N/A
Small ribosomal subunit protein bS18	rpsR	A0A060QIA3	No	N/A
Dipeptidyl-peptidase		A0AAN4U3J0	No	N/A
ABC transporter ribose permease		A0A0P0YG43	No	N/A
Uncharacterized protein		A0AAN4U370	No	N/A
Large ribosomal subunit protein uL6	rplF	A0A060QL77	No	N/A
Peptidase S41		A0AAN4U2A6	No	N/A
Small ribosomal subunit protein uS14	rpsN	A0A060QG08	No	N/A
Glyoxalase/bleomycin resistance protein/dioxygenase		A0A0P0YE78	No	N/A
Ribitol 2-dehydrogenase		A0A060QD11	No	N/A
Small ribosomal subunit protein uS5	rpsE	A0A060QGL7	No	N/A
Large ribosomal subunit protein uL15	rplO	A0A060QHA1	No	N/A
Uncharacterized protein		A0AAN4R5Z1	No	N/A
2,3-Bisphosphoglycerate-independent phosphoglycerate mutase	gpml	A0AAN4R5A9	No	N/A
Small ribosomal subunit protein uS10	rpsJ	A0A060QHC3	No	N/A
Nucleoside hydrolase		A0AAN4R136	No	N/A
Short-chain dehydrogenase		A0AAN4R3C5	No	N/A
Oxidoreductase		A0AAN4U3L9	No	N/A
Serine hydroxymethyltransferase 2	glyA 2	A0AAN4R3R0	No	N/A
Uncharacterized protein		A0AAN4R1T3	No	N/A
RND transporter		A0AAN4U3N7	No	N/A
Large ribosomal subunit protein uL24	rplX	A0A433WZC1	No	N/A
Methylmalonate-semialdehyde dehydrogenase (CoA acylating)		A0A060QD80	No	N/A
Uncharacterized protein		A0AAN4R3S1	No	N/A
Branched-chain amino acid aminotransferase		A0A0N7KUJ6	No	N/A
Ribose 5-phosphate isomerase B		A0A060QKL8	No	N/A
Methyltransferase		A0AAN4R2T7	No	N/A
Large ribosomal subunit protein uL2	rplB	A0A060QG18	No	N/A
Bacterioferritin		A0A060QM49	No	N/A
Fructose-bisphosphate aldolase		A0A060QIK2	No	N/A
Oxidoreductase		A0AAN4R2I8	No	N/A
UDP-N-acetylglucosamine 1-carboxyvinyltransferase	murA	A0AAN4R5D3	No	N/A
Oxidoreductase		A0AAN4R134	No	N/A
Arylesterase		A0AAN4R2P4	No	N/A
RND transporter MFP subunit		A0AAN4R2W3	No	N/A
Pyridine nucleotide-disulfide oxidoreductase		A0AAN4U2K0	No	N/A
Trigger factor	tig	A0AAN4R4C8	No	N/A
O-succinylhomoserine sulfhydrylase		A0A0P0YHT2	No	N/A
Uncharacterized protein		A0AAN4R119	No	N/A
Large ribosomal subunit protein uL1	rplA	A0A060QL87	No	N/A
Histone-like DNA-binding protein		A0A060QMB0	No	N/A
Thioredoxin	trxA	A0AAN4U2T7	No	N/A
Small ribosomal subunit protein uS9	rpsI	A0A060QCF7	No	N/A
Glucose 1-dehydrogenase		A0A060QH73	No	N/A
Adenosine kinase		A0AAN4R1Q4	No	N/A
3-Isopropylmalate dehydrogenase	leuB	A0AAN4R3T0	No	N/A
Co-chaperonin GroES	groES	A0A060QG87	No	N/A
Oxidoreductase		A0AAN4R261	No	N/A
Uncharacterized protein		A0AAN4R504	No	N/A
Large ribosomal subunit protein uL23	rplW	A0A060QGN7	No	N/A
Ribose-5-phosphate isomerase A	rpiA	A0AAN4R140	No	N/A
Iron transporter		A0AAN4R551	No	N/A
Elongation factor G	fusA	A0A060QDU2	No	N/A
Carbonic anhydrase		A0AAN4R3D6	No	N/A
Large ribosomal subunit protein uL3	rplC	A0A060QL83	No	N/A
Large ribosomal subunit protein uL5	rplE	A0A060QGM4	No	N/A
NAD(P)-dependent oxidoreductase		A0AAN4R2F6	No	N/A
Glucose-1-phosphate thymidylyltransferase		A0AAN4R444	No	N/A
Small ribosomal subunit protein bS21	rpsU	A0A060QED0	No	N/A
30S ribosomal protein S13	rpsM	A0AAN4R629	No	N/A
Ribosome-recycling factor	frr	A0AAN4R1F0	No	N/A
Ketol-acid reductoisomerase [NADP(+)]	ilvC	A0AAN4R241	No	N/A
50S ribosomal protein L25	rplY	A0AAN4U470	No	N/A
Uncharacterized protein		A0AAN4U1R5	No	N/A
Electron transfer flavoprotein subunit alpha	etfA	A0AAN4U3L0	No	N/A
ATP synthase subunit beta	atpD	A0A060QGJ6	No	N/A
Large ribosomal subunit protein uL16	rplP	A0A433WZ37	No	N/A
2,3,4,5-Tetrahydropyridine-2,6-dicarboxylate N-succinyltransferase	dapD	A0A060QGG0	No	N/A
50S ribosomal protein L10	rplJ	A0AAN4R616	No	N/A
Uncharacterized protein		A0AAN4R5I3	No	N/A
CarD-like transcriptional regulator		A0A060QFU4	No	N/A
Nitroreductase		A0AAN4R608	No	N/A
AtsE protein		A0A060QDR1	No	N/A
Ubiquinol oxidase subunit 2	cyoA-2	A0AAN4U2F9	No	N/A
Small ribosomal subunit protein uS4	rpsD	A0A060QGK9	No	N/A
Glucokinase	glk	A0AAN4R618	No	N/A
Small ribosomal subunit protein uS7	rpsG	A0A060QGP2	No	N/A
Alkyl hydroperoxide reductase subunit F		A0AAN4R2R0	No	N/A
Orotate phosphoribosyltransferase	pyrE	A0AAN4R0P4	No	N/A
Small ribosomal subunit protein uS3	rpsC	A0A060QKP6	No	N/A
Large ribosomal subunit protein bL9	rplI	A0A060QGW6	No	N/A
Aldo/keto reductase		A0A0P0YDI1	No	N/A
Polyribonucleotide nucleotidyltransferase	pnp	A0AAN4R3B1	No	N/A
50S ribosomal protein L4	rplD	A0AAN4U2L2	No	N/A
Adenosylhomocysteinase	ahcY	A0AAN4U3J2	No	N/A
6,7-Dimethyl-8-ribityllumazine synthase	ribH	A0A060QCI0	No	N/A
Uncharacterized protein		A0AAN4R4U2	No	N/A
Small ribosomal subunit protein uS11	rpsK	A0A060QFZ7	No	N/A
Dehydrogenase		A0AAN4R3U1	No	N/A
4-Hydroxy-tetrahydrodipicolinate synthase	dapA	A0AAN4R671	No	N/A
Two-component response regulator		A0AAN4R4X7	No	N/A
Ornithine carbamoyltransferase	argF	A0AAN4R2U3	No	N/A
Class A beta-lactamase		A0A0P0YGZ0	No	N/A
Succinate-semialdehyde dehydrogenase		A0AAN4R0B7	No	N/A
Protein-export protein SecB	secB	A0A060QC78	No	N/A
Dihydrolipoyl dehydrogenase		A0AAN4R451	No	N/A
Acyl carrier protein	acpP	A0A060QG03	No	N/A
Adenylosuccinate synthetase	purA	A0A060QDG3	No	N/A
Ribokinase	rbsK	A0AAN4U4C7	No	N/A
Gluconate 5-dehydrogenase		A0AAN4QZZ2	No	N/A
Secretion protein HlyD		A0AAN4R3X2	No	N/A
Small ribosomal subunit protein uS8	rpsH	A0A060QHA7	No	N/A
Large ribosomal subunit protein bL12	rplL	A0A060QGP9	No	N/A
Aminotransferase		A0A060QK27	No	N/A
2-Dehydro-3-deoxyphosphogluconate aldolase/4-hydroxy-2-oxoglutarate aldolase		A0A0P0YGS3	No	N/A
Inorganic pyrophosphatase	ppa	A0AAN4R535	No	N/A
Cold shock proein		A0A060QCT0	No	N/A
Uncharacterized protein		A0AAN4U3T1	No	N/A
2-Hydroxy-3-oxopropionate reductase	garR	A0AAN4U2B7	No	N/A
Tryptophan synthase alpha chain	trpA	A0A060QJC2	No	N/A
Dihydrolipoyl dehydrogenase		A0AAN4R1C6	No	N/A
Farnesyl-diphosphate synthase		A0AAN4U2K2	No	N/A
Putative ABC transporter, periplasmic sugar binding protein		A0A060QCK3	No	N/A
Dihydrolipoyllysine-residue succinyltransferase component of 2-oxoglutarate dehydrogenase complex	odhB	A0AAN4R2E8	No	N/A
RND transporter		A0AAN4U2D0	No	N/A
Acetate/propionate family kinase		A0A0P0YGF8	No	N/A
AdeC/adeK/oprM family multidrug efflux complex outer membrane factor	cusC	A0AAN4U2Q1	No	N/A
Small ribosomal subunit protein uS15	rpsO	A0A060QKD5	No	N/A
ABC transporter substrate-binding protein		A0A0P0YFG6	No	N/A
Ribonuclease PH	rph	A0AAN4R3P2	No	N/A
Serine protease		A0AAN4U477	No	N/A
Uncharacterized protein		A0AAN4U398	No	N/A
Small ribosomal subunit protein bS6	rpsF	A0A060QCT5	No	N/A
Oxidoreductase/SDR, 3-oxoacyl-[acyl carrier protein] reductase		A0A0P0YFM9	No	N/A
Orotidine 5'-phosphate decarboxylase	pyrF	A0AAN4R749	No	N/A
Protein GrpE	grpE	A0AAN4R3P3	No	N/A
Uncharacterized protein		A0AAN4R0M9	No	N/A
Beta-ketoacyl-ACP reductase		A0AAN4U2I6	No	N/A
dCTP deaminase	dcd	A0A060QDS5	No	N/A
Phosphate acetyl/butaryl transferase		A0A0P0YG52	No	N/A
Cysteine synthase A		A0AAN4R262	No	N/A
Amidohydrolase		A0A0P0YIA8	No	N/A
Lipoprotein		A0AAN4U1Y1	No	N/A
D-2-hydroxyacid dehydrogenase		A0AAN4R453	No	N/A
Inositol monophosphatase		A0AAN4R5J4	No	N/A
Phosphoribosylformylglycinamidine synthase subunit PurQ	purQ	A0A060QGZ2	No	N/A
Uncharacterized protein		A0AAN4U2X0	No	N/A
Citrate synthase		A0A060QBT6	No	N/A
Phosphoribosylformylglycinamidine synthase subunit PurL	purL	A0AAN4R4N2	No	N/A
Aminopeptidase		A0AAN4R1C3	No	N/A
MBL fold hydrolase		A0AAN4U3W7	No	N/A
Small ribosomal subunit protein uS2	rpsB	A0A060QJ90	No	N/A
Response regulator in two-component regulatory system with PhoQ		A0A060QIV1	No	N/A
Thioredoxin		A0AAN4R102	No	N/A
Nitrogen regulatory protein P-II		A0A060QII8	No	N/A
Phosphoribosylamine-glycine ligase	purD	A0AAN4R362	No	N/A
Uncharacterized protein		A0AAN4U2U4	No	N/A
Pyridoxine/pyridoxamine 5'-phosphate oxidase	pdxH	A0AAN4U1L8	No	N/A
Lactoylglutathione lyase		A0A060QBR9	No	N/A
GMP synthase [glutamine-hydrolyzing]	guaA	A0A060QC18	No	N/A
Alcohol dehydrogenase	dhaT	A0AAN4U3E3	No	N/A
Histidinol dehydrogenase	hisD	A0AAN4U3N9	No	N/A
Translation initiation factor IF-2	infB	A0AAN4U1D2	No	N/A
DNA-binding protein HU-beta		A0A060QJ81	No	N/A
Uncharacterized protein		A0AAN4R454	No	N/A
Unknown function DUF461 domain protein		A0A0P0YFU8	No	N/A
Cytosol aminopeptidase	pepA	A0AAN4R3L5	No	N/A
NADH-ubiquinone predicted oxidoreductase chain E		A0A060QJS7	No	N/A
Amidophosphoribosyltransferase	purF	A0AAN4U3Q0	No	N/A
GTP-binding protein		A0AAN4R246	No	N/A
50S ribosomal protein L30	rpmD	A0AAN4R2I0	No	N/A
dTDP-glucose 4,6-dehydratase		A0A0P0YDI0	No	N/A

^*a*^ N/A: no BastionHub prediction for secretion.

^*b*^ Gene name cells are empty if there is no available annotated gene name.

**TABLE 4:** Proteins predicted to be secreted through T1SS and T4SS

Protein name	Accession	Media	Predicted secretion system	BastionHub prediction results
DUF3309 domain-containing protein	A0A433WW75	Chocolate	I	0.807
Bulb-type lectin domain-containing protein	A0AAN4U1B4	Minimal	I	0.972
Uncharacterized protein	A0AAN4R2X5	Minimal	I	0.756
Cytoplasmic protein	A0A060QI69	Minimal	IV	0.919
Outer membrane protein	A0AAN4U3I6	Both	I	1
Flagellar hook protein FlgE	A0AAN4R3W6	Both	I, II	45,292
Lipoprotein	A0AAN4U3B7	Both	I	0.924
Outer membrane protein	A0AAN4R2L9	Both	I	1
Lipoprotein	A0A060QD82	Both	I	0.997
UrcA family protein	A0AAN4R0P5	Both	IV	0.716
Hypervirulence associated protein TUDOR domain-containing protein	A0A060QID8	Both	IV	0.622

All proteins were entered into The UniProt Consortium (https://www.uniprot.org/) to identify the most recently updated accession numbers, protein names, and gene names, if available (22). The InterPro database was used to identify protein domains, family names, if available, and Gene Ontology (GO) terms for molecular function and biological process (23). Proteins predicted to be secreted by the BastionX software were inputted into BlastKOALA to functionally characterize and automatically annotate the known gene products (24, 25). All graphs were made using GraphPad Prism version 10.4.1 for Windows (GraphPad Software, Boston, Massachusetts USA; https://www.graphpad.com/).

## RESULTS

### Proteins identified under all growth conditions

There were 343 proteins identified from all samples. Eighty-two proteins were uniquely identified from the samples grown on minimal media, while 32 proteins were uniquely identified from the samples grown on chocolate agar (Tables 1 and 2). Proteins uniquely found in each condition were further analyzed using BastionX to predict whether they are likely to be secreted from *A. bogorensis*. Of the 82 proteins identified from the minimal media samples, 19 of these were predicted to be secreted. Twenty-two of the 32 proteins uniquely identified from the chocolate agar samples were predicted to be secreted (Tables 1 and 2). One of the proteins identified was a peptide less than 50 amino acids long and could not be analyzed by the BastionHub software. This was identified as a DUF333 domain-containing protein on UniProt. One hundred six of the 343 proteins identified under both growth conditions were predicted to be secreted by BastionHub (Table 3).

A total of 8 proteins were predicted to be secreted through the T1SS, 76 through the T2SS, 43 through the type III secretion system (T3SS), 3 through the T4SS, and 21 through the T6SS. The majority of the identified proteins were not predicted to be secreted (Tables 1 through 3). Four proteins predicted to be secreted were equally likely to be secreted through two secretion systems (Tables 1 and 3).

**Fig 1:**
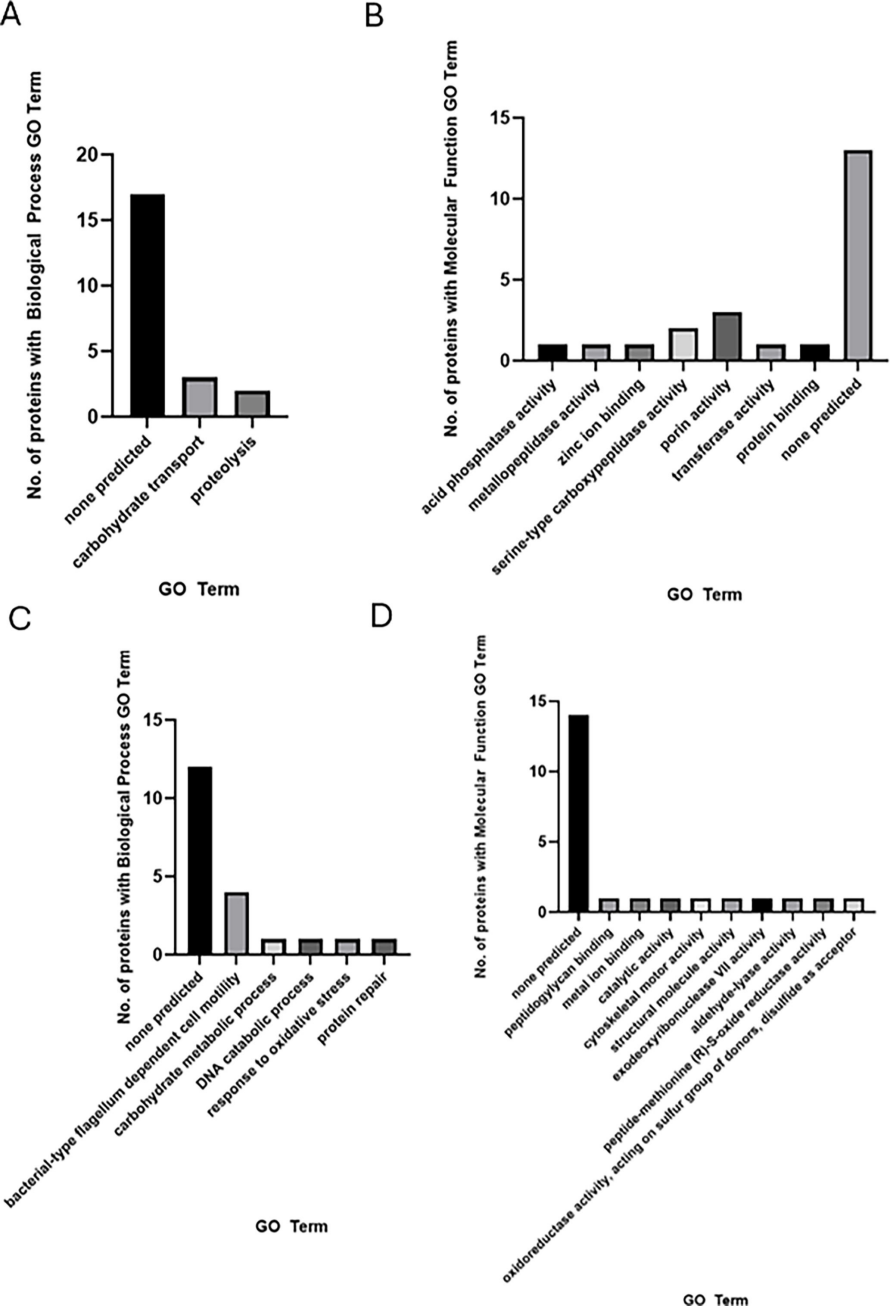
GO terms associated with proteins predicted to be secreted on minimal and chocolate agar. (A, B) GO terms associated with proteins predicted to be secreted under chocolate agar conditions. (A) Biological process GO terms. (B) Molecular function GO terms. (C, D) GO terms associated with proteins predicted to be secreted only under minimal media conditions. (C) Biological process GO terms. (D) Molecular function GO terms.

**Fig 2:**
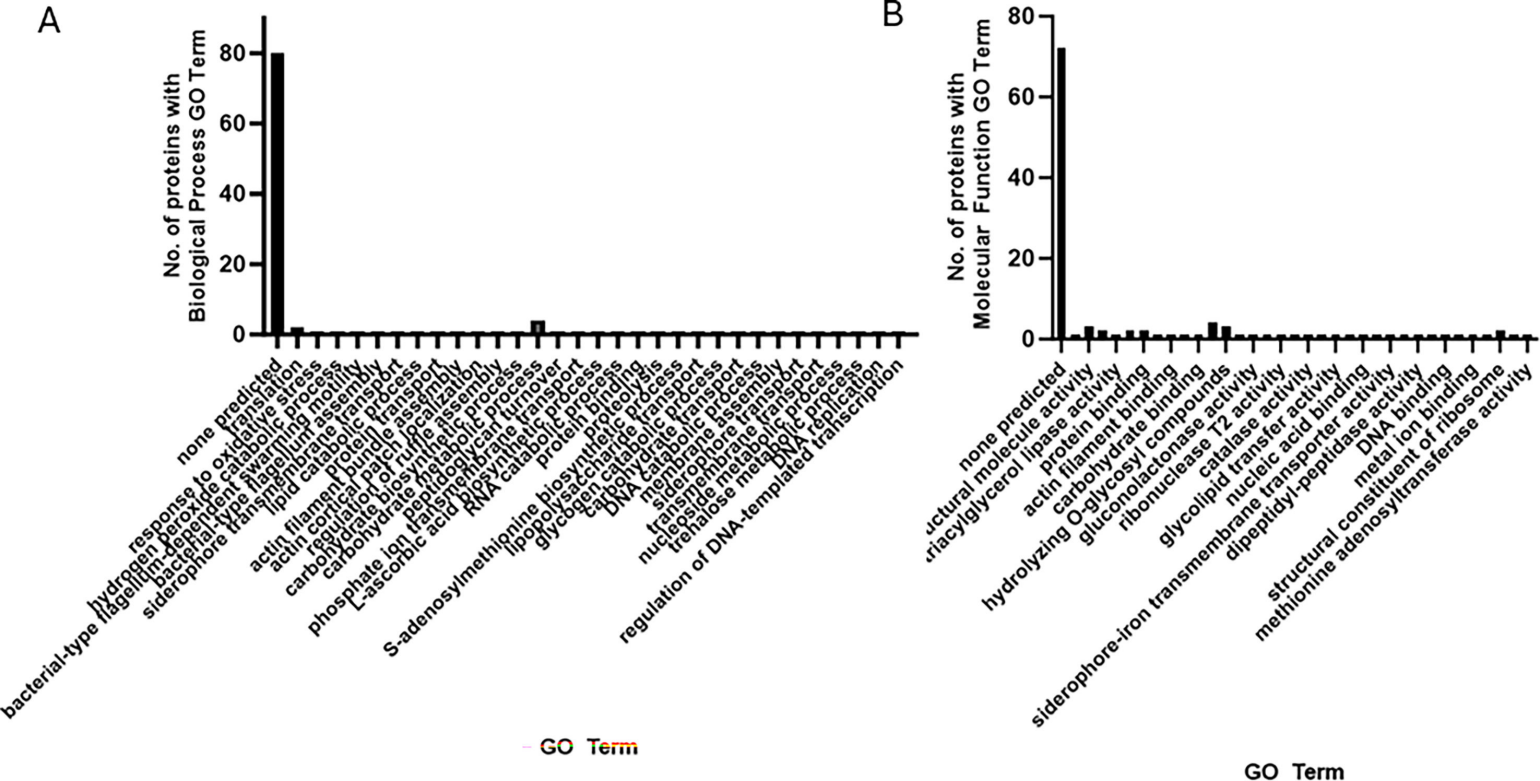
GO terms associated with proteins predicted to be secreted under both growth conditions. (A, B) GO terms associated with proteins predicted to be secreted under both chocolate and minimal media conditions. (A) Biological process GO terms. (B) Molecular function GO terms.

When available, we annotated the molecular function and biological process GO terms for the proteins using the InterPro database, as shown in Table S1 through S3. Most of the proteins have no predicted molecular function or biological process. Sorted GO terms are available in Table S4 through S6. There were eight unique biological process-associated GO terms and 21 unique GO terms characterizing molecular function for proteins identified on chocolate agar. Forty-nine GO terms were associated with biological process, and 75 GO terms were annotated for proteins identified on minimal media agar. Under both conditions, 99 unique GO terms were associated with biological process, and 170 unique GO terms were annotated for molecular function. All GO terms associated with proteins predicted to be secreted by BastionX are shown in Fig. 1 and 2.

### Proteins predicted to be secreted through the T1SS

Eight total proteins were predicted to be secreted through the T1SS across both growth conditions. A single protein was predicted to be secreted through the T1SS on chocolate agar. This is a DUF3309 domain-containing protein of unknown function with a helical domain annotated as a probable membrane or transmembrane protein and a signal peptide. Two proteins were predicted to be secreted through the T1SS under minimal media growth conditions. One is a bulb-type lectin domain-containing protein, 745 amino acids in length, with no other information known. The other is an uncharacterized protein of unknown function. The other five proteins were identified under both conditions. The most well-characterized protein identified is flagellar hook protein FlgE. This protein belongs to the flagellar hook-basal body protein FlgE/F/G family. This protein links the flagellar filament to the drive apparatus in the basal body (Table 4). Two of these are outer membrane proteins, one of which, A0AAN4U3I6, is involved in binding calcium ions. There is nothing else known about either protein. There are also two lipoproteins predicted to be secreted under both conditions. The first, accession A0AAN4U3B7, has an annotated putative signal peptide. The other is accession A0A060QD82, 91 amino acids in length, with an annotated disordered region and no other characterization.

### Proteins predicted to be secreted through T4SS

No proteins were predicted to be secreted through the T4SS solely when grown on chocolate agar. One protein, identified as a cytoplasmic protein, was found under minimal media growth conditions and was predicted to be secreted through the T4SS (Table 4). This protein is 194 amino acids in length and a member of the transcriptional cell cycle regulator TrcR family. No other information is known about the protein. Two proteins were identified on both chocolate agar and minimal media growth conditions. One is a UrcA family protein with a signal peptide. No other structural or functional information is known. The last is a hypervirulence-associated protein TUDOR domain-containing protein. Along with this domain, there is also a disordered region. No other characterization for this protein is available.

### Proteins across all growth conditions are involved in similar processes

**Fig 3:**
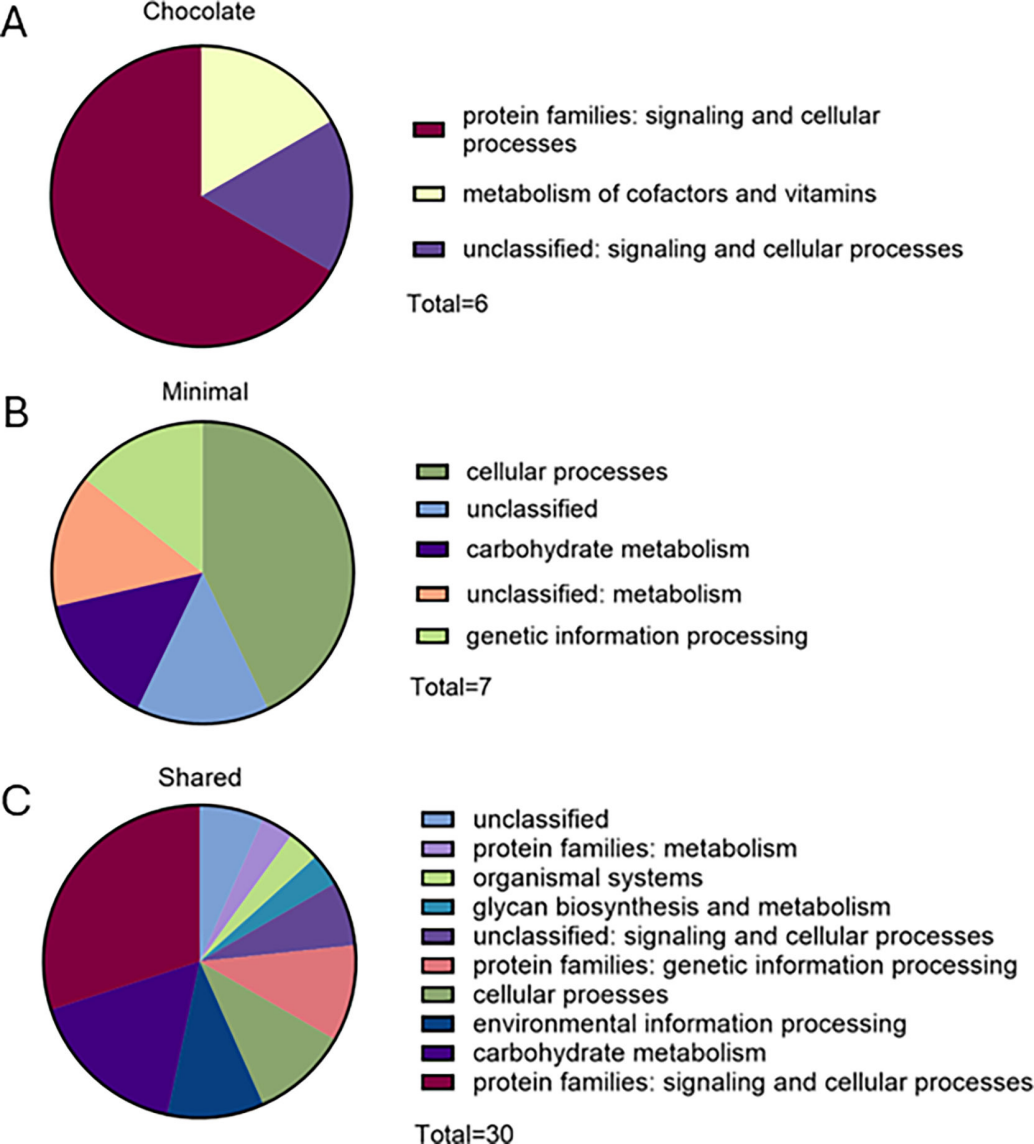
Proteins predicted to be secreted were classified as belonging to a range of protein families and functional groups. All proteins identified under each growth condition that were also predicted to be secreted by the BastionX software were entered into the BlastKOALA server as FASTA files. (A) Proteins identified only on chocolate agar media. (B) Proteins identified only on minimal agar media. (C) Proteins identified under both growth conditions.

A total of six proteins secreted under chocolate agar conditions were annotated by the BlastKOALA automatic annotation and characterization software. Four of these are members of protein families involved in signaling and cellular processes (accession A0A060QDK6, A0AAN4R463, A0A060QGH0, A0A060QIF4). One is involved in signaling and cellular processes (accession A0A060QKU7), and the last is characterized as involved with metabolism of cofactors and vitamins (accession A0AAN4U1B7) (Fig. 3A).

Seven proteins identified on minimal media only and predicted to be secreted were annotated by the BlastKOALA software. Three of these proteins are functionally characterized as involved in cellular processes (accession A0A060QIM2, A0AAN4R3E9, A0A060QIL6). One protein is involved in carbohydrate metabolism (accession A0AAN4U2Z8). One of these proteins is involved in genetic information processing (accession A0A060QFS4), one is characterized as unclassified: metabolism (accession A0A060QFX0), and one protein was annotated by the program but was unclassified (accession A0A060QI69) (Fig. 3B). Thirty proteins predicted to be secreted under both conditions were annotated by the KEGG/BlastKOALA software. Ten proteins were classified as belonging to protein families involved with signaling and cellular processes (accession A0AAN4U233, A0AAN4R251, A0AAN4R0H1, A0AAN4R6T1, A0AAN4R312, A0AAN4R021, A0AAN4R0C6, A0AAN4U3E7, A0AAN4R5L8, A0AAN4R4Z7). Five of the proteins are involved in carbohydrate metabolism (accession A0AAN4R0F1, A0AAN4R2Y8, A0A060QIY2, A0AAN4R1X4, A0AAN4U3F6). Three of the proteins were classified as proteins belonging to families involved in genetic information processing (accession A0AAN4R2R6, A0AAN4R1H4, A0AAN4R537), while three proteins were also identified to relate to environmental information processing (accession A0AAN4R5R2, A0AAN4R2W7, A0AAN4R3M9). Two of these proteins are involved in cellular processes (accession A0AAN4R3W6, A0A060QGH4), and two of these proteins were annotated as unclassified: signaling and cellular processing (accession A0A060QIL1, A0AAN4R498). Two proteins were annotated as belonging to protein families involved with metabolism (accession A0AAN4U1L7, A0AAN4U369), and one was characterized to be involved in glycan biosynthesis and metabolism (accession A0A060QKA2). Two proteins were annotated as unclassified proteins of unknown function (accession A0AAN4U2U5, A0AAN4R4K7) (Fig. 3C).

## DISCUSSION

This is the first study to identify proteins released by *A. bogorensis*. The genome of *A. bogorensis* has been sequenced, but there is little to no experimental evidence of the proteins produced by the bacterium under different growth conditions. All proteins identified in this work were inferred based on relationships to homologs in closely related species or predicted to exist without any experimental evidence at the protein, transcript, or homology levels (22). A total of 32 proteins unique to chocolate agar, 82 proteins unique to minimal agar, and 343 proteins secreted under both conditions were identified using liquid chromatography with tandem mass spectrometry. We were able to predict the secretion of 19 proteins under minimal media growth conditions and 22 proteins that were predicted to be secreted under blood meal-like conditions. These proteins were predicted to be secreted through the type I, II, III, IV, and VI secretion systems (16).

Many of the proteins identified were not predicted to be secreted by the bacterium and instead likely function in the cytoplasm. Due to the growth time of the bacteria prior to sample collection, it is probable that some cell death occurred, leading to protein release as the cells lysed. This lysis can explain the high number of intracellular proteins identified here. Despite this, we still identified a considerable number of proteins that were predicted to be secreted under each growth condition and predicted the mode of transport out of the cell for each. We understand that this study does have some limitations. This is not a quantitative study and instead measures only the presence of proteins under each growth condition. Continuing this work with a focus on the proteins identified under all growth conditions may lead to the discovery of genes that are differentially expressed under each growth condition. Future work could be done to elucidate this further. Furthermore, many of the proteins identified here are uncharacterized or inferred based on homology to those in other bacterial species. Future studies could focus on a small subset of these proteins to better understand how these proteins function. However, this study represents a significant start toward understanding what molecules are released by *A. bogorensis*, especially under conditions relevant for improving paratransgenesis.

The T1SS is a one-step translation system in gram-negative bacteria that spans the cell envelope and transports proteins without a periplasmic intermediate. The substrates of this secretion system are monomeric unfolded proteins that are either destined for the cell surface or extracellular milieu and often function as adhesins, lipases, proteases, heme-binders, and toxins (1, 26, 27).

In contrast, the type II secretion system is more complex. While it also exports proteins to the cell surface and extracellular environment, this secretion system transports both monomeric and multimeric proteins using a two-step translocation system (28). In this case, unfolded substrates are transported to the periplasm, where they fold. Once folded, they are transported outside of the cell. Many of the substrates secreted through the T2SS function as toxins and biofilm components, but many are lipases and other lytic enzymes (1, 15).

The T3SS is also a one-step translocation system but differs from T1SS in that it has a secretion channel through which a needle complex extrudes; the outer membrane channel also has rings for flagella assembly. Substrates are destined for either flagellum assembly or for injection into eukaryotic cells (29, 30). Found in gram-negative, gram-positive, and archaea, the type IV secretion system is widely distributed, with secreted proteins often destined for other bacterial or eukaryotic cells as well as the extracellular milieu or conjugative pili (7, 31). The T4SS also utilizes direct transport of effector proteins to target cells. Proteins secreted through this pathway are often involved in pathogenicity and infection. The type VI secretion system is unique to gram-negative bacteria and transports substrates to bacterial or eukaryotic cells. These substrates function primarily as effectors for eukaryotic cell processes, and antibacterial and anti-eukaryotic toxins (32, 33).

We hypothesize that using T1SSs or T4SSs for secreting paratransgenic effectors will improve secretion and result in increased bacterial fitness. Several groups have already tested and shown efficient secretion of anti-*Plasmodium* effectors through T1SSs, such as the well-characterized native *Escherichia coli* hemolysin A (HlyA) secretion system and the native *Serratia marcescens* HasA (heme-binding protein) system. Bisi and Lampe engineered *Pantoea agglomerans* to secrete anti-*Plasmodium* effector molecules using the *E. coli* HlyA type I secretion signal. *P. agglomerans* successfully secreted HlyA-effector fusions, and the strains grew as efficiently as wild-type strains under laboratory conditions (34). Wang et al. evaluated *P. agglomerans* strains expressing several anti-*Plasmodium* effector molecules secreted through the HlyA system and found that they reduced *P. berghei* and *Plasmodium falciparum* incidence and prevalence in *Anopheles* mosquitoes significantly (35). In 2017, Wang and colleagues isolated and genetically modified a *Serratia* strain to secrete anti-*Plasmodium* effectors using the native HasA secretion system (36). The strains secreted high levels of the effectors and were able to significantly inhibit *Plasmodium* development in *Anopheles* mosquitoes, showcasing the feasibility of utilizing T1SSs for paratransgenesis. We propose using the data described here to improve anti-*Plasmodium* effector secretion by *A. bogorensis*. We suggest using secretion signals from some of the type I-secreted or type IV-secreted proteins identified in this work. Specifically, we propose to use these secretion signals to direct the anti-parasitic effector molecules out of paratransgenic *A. bogorensis* strains. As described earlier, successful paratransgenesis systems rely on efficient secretion of anti-parasitic molecules in the vector organism (15, 37, 38). The anti-parasitic effector molecule cannot optimally target the parasite without efficient secretion. Researchers have engineered *A. bogorensis* paratransgenic strains that secrete the antimicrobial peptide scorpine modified with T2SS signal sequences (15, 37). While several of these signal sequences improved extracellular levels of scorpine while also preventing *P. berghei* development in the mosquito midgut, the paratransgenic strains were significantly less fit than the wild-type *Asaia* strain. For this reason, we expect that utilizing the T1SS instead of the T2SS for anti-*Plasmodium* effector secretion will not only improve secretion but may also improve the fitness and survivability of the paratransgenic *Asaia* strains in future work. We also hypothesize that using T4SSs for paratransgenesis would improve secretion and strain fitness. Anti-parasitic effectors could be directed out of the cell directly into *Plasmodium* cells (1, 7). This would avoid any effector presence in the extracellular milieu and may result in better *Plasmodium* reduction than secretion of effectors into the midgut environment.

### A proposal for minimalistic paratransgenesis

**Fig 4:**
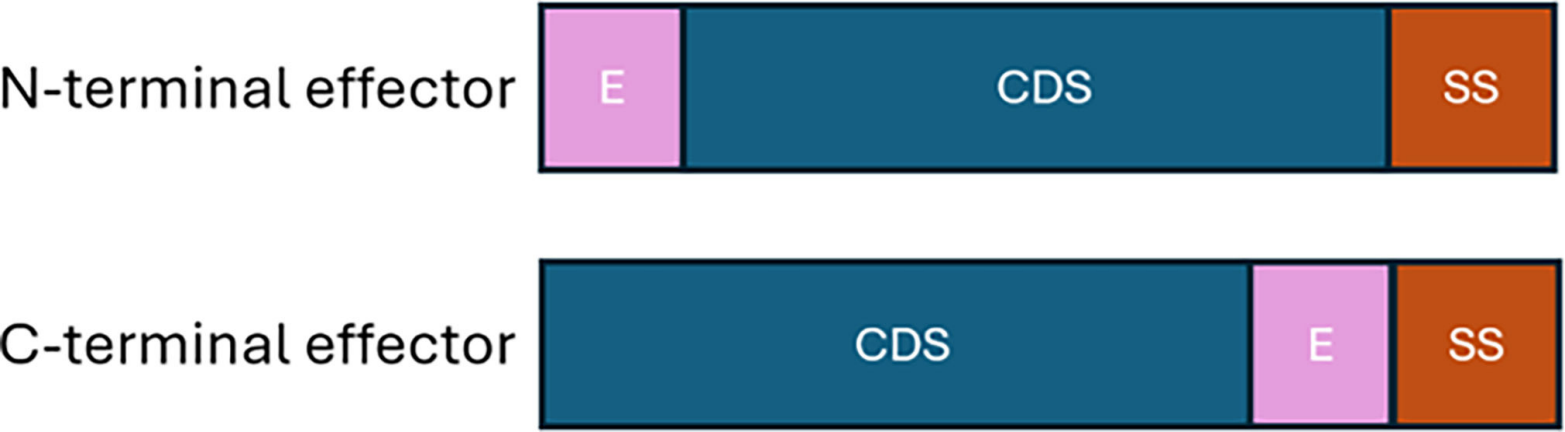
Minimal paratransgenic strain chromosome. Linearized chromosomal fragment. Genes for the effector peptide (EPIP) can be fused to the coding sequence for blood meal-inducible secreted proteins and immediately upstream or downstream from the T1-secreted protein. CDS, substrate coding sequence; SS, type I signal sequence; E, effector sequence.

One potential use of these data is the creation of a minimal paratransgenesis system. While more complicated plasmid-based paratransgenesis strains exist and are currently being developed, we propose utilizing these single-step secretion systems (types I and IV) to engineer genetically simple and effective alternatives that only require insertion of anti-*Plasmodium* effector genes into the chromosome of the bacterium. Instead of requiring plasmids and antibiotic resistance mechanisms for strain maintenance, a small effector coding sequence can be inserted into the chromosome of *A. bogorensis* directly upstream of the T1SS protein signal of interest or as an N-terminal fusion. For example, the effector could be inserted in between the protein-coding locus and the secretion signal. This way, the native secretion signal can be utilized to facilitate effector secretion. A simple illustration of this is shown in Fig. 4. Insertion of small effector peptides, such as enolase-plasminogen interaction peptide (EPIP) or midgut peptide 2, which are 11 amino acids and 12 amino acids, respectively, may also help to preserve the function of the protein encoded at the wild-type locus and still be easily secreted fused to the native type I-secreted protein (35, 39, 40).

This minimal paratransgenesis system would have several advantages compared to plasmid-based strains published to date. Desirable properties of any paratransgenesis system are conditional expression of the anti-parasitic effector, secretion of the effector in sufficient amounts, genetic stability, and minimal fitness costs to the bacterial strain. The minimal system we propose here may fulfill these requirements. First, insertion of the effector directly into the chromosome would enable more streamlined secretion. Using a protein that is secreted naturally in a blood meal environment, such as one of the candidates identified here, could allow for the conditional expression of this anti-*Plasmodium* effector. The effector could also be inserted directly downstream of a type I-secreted protein identified here under both growth conditions, allowing for its constitutive expression. The minimal paratransgenic locus would be more stable than the use of a plasmid-based system, as there would not be any reliance upon antibiotic resistance mechanisms for plasmid maintenance. Exogenous DNA is inherently unstable if not selected for, and bacteria can readily transfer plasmids through conjugation (41, 42). As such, insertion of the anti-*Plasmodium* effector would be more stable and unlikely to be lost. These strains may also be field safe, as there would also be no chance of release of antibiotic resistance genes to unintended organisms in the environment. Finally, we expect this minimal system to have fewer fitness defects than the more complex plasmid-based strains. This is because there would be no extra genes to express, and the paratransgenic effector peptide would be released as part of the native protein. These minimal paratransgenic strains could be a simple next step toward preventing human malaria.

## Data Availability

The raw LC-MS/MS data are available at the Dryad Digital Repository. The Dryad data set is titled "Raw dataset for a catalogue of proteins released from *Asaia bogorensis* under two growth conditions" and is available at https://doi.org/10.5061/dryad.4mw6m90nd.

## SUPPLEMENTAL MATERIAL

Supplemental tables spectrum.01506-25-s0001.docx Tables S1 to S7.

ASM does not own the copyrights to Supplemental Material that may be linked to, or accessed through, an article. The authors have granted ASM a non-exclusive, world-wide license to publish the Supplemental Material files. Please contact the corresponding author directly for reuse.
