# Supplementary material for: A catalog of proteins released from Asaia bogorensis under two growth conditions
Source: Microbiol Spectr. 2025 Aug 29;13(10):e01506-25. doi: 10.1128/spectrum.01506-25 (PMC12502724; doi:10.1128/spectrum.01506-25)
Supplement: Supplemental tables — Tables S1 to S7. [file spectrum.01506-25-s0001.docx]

Supplemental Table 1. Proteins identified on chocolate media agar and all secretion predictions Gene Ontology Terms Gene Ontology Terms

Protein Name Gene Name Accession

Predicted Secretion System

BastionHub Prediction

Results Molecular Function Biological Process

TonB-dependent receptor A0A060QDK6 I, II 0.538, 0.762 none predicted none predicted

DUF3309 domain-containing protein A0A433WW75 I 0.807 none predicted none predicted

Signal peptidase A0AAN4R151 I, III, IV, VI 0.61, 0.924, 0.866, 0.578 none predicted none predicted

Hypothetical protein A0A0P0YDV3 I, II, III, IV, VI 0.793, 0.692, 1, 0.857, 0.717 none predicted none predicted Acid phosphatase phoC A0AAN4U1B7 II 0.681 acid phosphatase activity none predicted Carboxypeptidase-related protein A0A060QFN3 II, VI 1, 0.901 serine-type carboxypeptidase activity proteolysis Peptidase A0AAN4R7N1 II, VI 0.973, 0.82 metallopeptidase activity, zinc ion binding none predicted

Lipoprotein A0A060QI71 II, III, VI 0.685, 1, 0.816 none predicted none predicted

outer membrane lipoprotein omlA A0AAN4R4A8 II 0.535 none predicted none predicted

carbohydrate-selective porin A0AAN4R463 II, VI 0.956, 0.729 porin activity carbohydrate transport

carbohydrate-selective porin A0A060QGH0 II, VI 1, 0.85 porin activity carbohydrate transport Peptidase S10 A0AAN4R155 II, VI 1, 0.727 serine-type carboxypeptidase activity proteolysis

DUF2272 domain-containing protein A0A060QD52 II, III, VI 1, 0.504, 1 none predicted none predicted

Porin A0A060QIF4 II, VI 1, 0.735 porin activity carbohydrate transport

Peptidase C51 domain-containing protein A0A060QLL6 II, VI 0.831, 0.83 none predicted none predicted

Cell wall-associated hydrolase A0AAN4R1B4 II, VI 45292 none predicted none predicted

Putative lipoprotein A0A060QKU7 II 0.81 none predicted none predicted

Uncharacterized protein A0AAN4R1Y5 II 0.653 none predicted none predicted

Lipoprotein A0A060QD84 II, III, VI 0.86, 0.781, 0.805 none predicted none predicted

Uncharacterized protein A0AAN4U2I1 III 0.984 protein binding none predicted

YkuD domain-containing protein A0A060QHE7 III, IV, VI 0.955, 0.563, 0.899 transferase activity none predicted

Lipoprotein A0A0P0YGH5 III 0.718 none predicted none predicted

DUF333 domain-containing protein A0AAN4R188 N/A N/A none predicted none predicted

lytic transglycosylase activity and hydrolase activity,

Lytic transglycosylase A0AAN4R3Q4 no N/A Putative dihydroxyacetone kinase,

dihydroxyacetone binding subunit A0A060QLZ6 no N/A

Probable periplasmic serine

hydrolyzing O-glycosyl compounds peptidoglycan metabolic process glycerone kinase activity and transferase activity,

transferring phosphorus-containing groups glycerol metabolic process

endoprotease DegP-like A0A060QHI0 no N/A protein binding and serine-type endopeptidase activity proteolysis

ribonucleoside triphosphate phosphatase activity

Alkaline phosphatase family protein A0AAN4R3Z5 no N/A and nucleoside triphosphate diphosphatase activity nucleoside triphosphate metabolic process

Putative cytochrome c-552 A0A060QBQ6 no N/A electron transfer activity and heme binding none predicted Biopolymer transport protein ExbB A0A060QGG7 no N/A none predicted protein import Lipopolysaccharide assembly protein

A domain-containing protein A0A060QJW1 no N/A none predicted none predicted

D-amino-acid oxidase activity, FAD binding,

D-amino-acid oxidase A0A060QKK3 no N/A oxidoreductase activity, flavin adenine dinucleotide binding D-amino acid metabolic process

Aspartate aminotransferase family protein A0AAN4U1J6 no N/A transaminase activity, pyridoxal phosphate binding none predicted

Supplemental Table 2. Proteins identified on minimal media agar and all secretion predictions Gene Ontology Terms Gene Ontology Terms

Predicted BastionHub Prediction

Protein Name Gene Name Accession Secretion System Results Molecular Function Biological Process

Uncharacterized protein A0AAN4R2X5 I, II, III 0.756, 0.773, 0.558 none predicted none predicted Bulb-type lectin domain-containing protein A0AAN4U1B4 I, II 0.972, 0.732 none predicted none predicted Pentapeptide MXKDX repeat protein A0A060QKH2 I, II, III, IV, VI 0.646, 0.669, 1, 0.908, 0.65 none predicted none predicted

Basal-body rod modification protein FlgD A0A060QIM2 I, II 0.776, 0.985 none predicted bacterial-type flagellum-dependent cell motility Flagellar basal-body rod protein FlgG A0AAN4R026 II 0.829 none predicted bacterial-type flagellum-dependent cell motility Sucrose isomerase/alpha amylase A0A0P0YFZ2 II 0.722 none predicted none predicted

SPOR domain-containing protein A0AAN4R683 II 0.601 peptidoglycan binding none predicted Cupin domain protein A0A0P0YDP2 II, VI 0.784, 1 none predicted none predicted

peptide-methionine (R)-S-oxide reductase activity,

oxidoreductase activity, acting on a sulfur group of

Peptide methionine sulfoxide reductase MsrB msrB A0A060QFX0 II, VI 0.507, 0.63 donors, disulfide as acceptor response to oxidative stress, protein repair

Gluconolactonase A0A060QJ35 II, VI 0.83, 0.675 none predicted none predicted cytoskeletal motor activity, structural molecule

Flagellar hook-basal body complex protein FliE fliE A0AAN4R3E9 III 0.7 activity bacterial-type flagellum-dependent cell motility

DUF3597 domain-containing protein A0A060QIH8 III 0.795 none predicted none predicted GcrA cell cycle regulator domain protein A0A060QHS5 III, IV, VI 0.769, 0.655, 0.64 none predicted none predicted Uncharacterized protein A0AAN4R2J3 III 0.582 none predicted none predicted

Flagellar basal body rod protein FlgB A0A060QIL6 III 0.838 none predicted bacterial-type flagellum-dependent cell motility Cytoplasmic protein A0A060QI69 III, IV 0.722, 0.919 none predicted none predicted

Exodeoxyribonuclease 7 small subunit xseB A0A060QFS4 III, IV 0.575, 0.518 exodeoxyribonuclease VII activity DNA catabolic process DUF4167 domain-containing protein A0A0P0YFQ5 III, IV 0.995, 0.75 none predicted none predicted Phosphoketolase A0AAN4U2Z8 VI 0.835 aldehyde-lyase activity, catalytic activity carbohydrate metabolic process 50S ribosomal protein L31 rpmE A0AAN4U452 no N/A structural constituent of ribosome translation

Electron transfer flavoprotein subunit beta etfB A0AAN4U493 no N/A electron transfer activity none predicted Uncharacterized protein A0AAN4R4B5 no N/A hydrolase activity none predicted

Peptidase C56 PfpI A0AAN4R1Z1 no N/A none predicted none predicted

Oxidoreductase A0AAN4R344 no N/A oxidoreductase activity none predicted

Trehalose 6-phosphate phosphatase ostB A0AAN4U3N5 no N/A trehalose-phosphatase activity trehalose biosynthetic process Acetylornithine aminotransferase A0A0P0YJ19 no N/A none predicted none predicted

Xanthine-guanine phosphoribosyltransferase gpt A0AAN4U365 no N/A xanthine phosphoribosyltransferase activity none predicted

ATP binding, nucleobase-containing compound kinase activity, adenylate kinase activity,

Adenylate kinase adk A0AAN4R1Q5 no N/A

phosphotransferase activity, phosphate group as acceptor

hydro-lyase activity, hydro-lyase activity, catalytic

nucleobase-containing compound metabolic process

Dihydroxy-acid dehydratase ilvD A0AAN4R3I2 no N/A activity branched-chain amino acid biosynthetic process

Phosphoribosylformylglycinamidine cyclo-ligase purM A0AAN4R0X8 no N/A phosphoribosylformylglycinamidine cyclo-ligase activit de novo' IMP biosynthetic process Thiol:disulfide oxidoreductase yfcG A0AAN4U3S2 no N/A disulfide oxidoreductase activity none predicted

Uncharacterized protein A0AAN4U262 no N/A none predicted none predicted Cell division protein FtsZ ftsZ A0AAN4U3Y8 no N/A GTP binding, GTPase activity cell division

magnesium ion binding, acireductone synthase

Enolase-phosphatase E1 mtnC A0AAN4R5T7 no N/A activity L-methionine salvage from methylthioadenosine

Phosphoglycerate mutase A0AAN4U3H2 no N/A phosphatase activity none predicted

nucleoside metabolic process, L-methionine

S-methyl-5'-thioadenosine phosphorylase mtnP A0AAN4R0K0 no N/A S-methyl-5-thioadenosine phosphorylase activity

dTDP-4-dehydrorhamnose 3,5-epimerase A0AAN4R541 no N/A dTDP-4-dehydrorhamnose 3,5-epimerase activity

salvage from methylthioadenosine polysaccharide biosynthetic process, dTDP- rhamnose biosynthetic process, extracellular polysaccharide biosynthetic process

Haloacid dehalogenase A0AAN4U2W3 no N/A hydrolase activity, phosphatase activity none predicted

L-methionine salvage from methylthioadenosine,

Methylthioribulose-1-phosphate dehydratase mtnB A0AAN4R296 no N/A metal ion binding, aldehyde-lyase activity pentose catabolic process

uncharacterized protein A0AAN4R1W2 no N/A none predicted none predicted

ATPase A0AAN4R216 no N/A ATP binding, ATP hydrolysis activity none predicted

uncharacterized protein A0AAN4U2H7 no N/A none predicted none predicted

Xylulose kinase xylB A0AAN4R5K4 no N/A

Glycosyl hydrolase A0AAN4R638 no N/A 3'(2'),5'-bisphosphate nucleotidase CysQ cysQ A0AAN4U2M7 no N/A

Ribulose-phosphate 3-epimerase rpe A0AAN4U269 no N/A

kinase activity, D-xylulokinase activity, phosphotransferase activity

hydrolase activity, hydrolyzing O-glycosyl compounds, beta-glucosidase activity

magnesium ion binding, 3'(2'),5'-bisphosphate nucleotidase activity

racemase and epimerase activity, acting on carbohydrates and derivatives, D-ribulose- phosphate 3-epimerase activity

carbohydrate metabolic process, xylulose metabolic process

carbohydrate metabolic process, glucan catabolic process

sulfur compound metabolic process, phosphatidylinositol phosphate biosynthetic process

carbohydrate metabolic process, pentose- phosphate shunt

glutathione biosynthetic process, cellular

Glutamate--cysteine ligase gsh1 A0AAN4R407 no N/A glutamate-cysteine ligase activity, catalytic activity modified amino acid biosynthetic process

Phosphoserine aminotransferase A0AAN4R1N4 no N/A

O-phospho-L-serine:2-oxoglutarate aminotransferase activity

DNA recombination, regulation of DNA-templated

L-serine biosynthetic process, glycine biosynthetic process

Integration host factor subunit beta ihfB A0A060QEZ9 no N/A

3-oxoacyl-[acyl-carrier-protein] synthase 2 fabF A0AAN4U2R1 no N/A GAF domain-containing protein A0A060QJ84 no N/A

transcription DNA binding, structural constituent of chromatin acyltransferase activity, transferring groups other

than amino-acyl groups, 3-oxoacyl-[acyl-carrier-

protein] synthase activity fatty acid biosynthetic process protein binding, L-methionine-(R)-S-oxide

reductase activity none predicted

Glutaredoxin A0AAN4R7C1 no N/A disulfide oxidoreductase activity none predicted RNA polymerase-binding transcription factor DksA dksA A0AAN4U273 no N/A zinc ion binding none predicted

Molybdenum cofactor biosynthesis protein B moaB A0AAN4R408 no N/A none predicted Mo-molybdopterin cofactor biosynthetic process peptidase T4 A0AAN4R010 no N/A aminopeptidase activity none predicted

protein peptidyl-prolyl isomerization, protein

Peptidyl-prolyl cis-trans isomerase A0AAN4R5V7 no N/A peptidyl-prolyl cis-trans isomerase activity

FMN binding, oxidoreductase activity, acting on NAD(P)H, quinone predicted or similar compound

folding

FMN-dependent NADH-azoreductase acpD A0AAN4R5X4 no N/A as acceptor none predicted

Adenine phosphoribosyltransferase apt A0AAN4U255 no N/A adenine phosphoribosyltransferase activity adenine salvage

thioredoxin-dependent peroxiredoxin A0A060QI59 no N/A oxidoreductase activity cellular response to oxidative stress NAD(P)-dependent oxidoreductase A0AAN4R1S4 no N/A oxidoreductase activity none predicted

phosphatase A0AAN4U2D8 no N/A

Histidinol-phosphatase A0AAN4R719 no N/A

glycerol-1-phosphatase activity, glycerol-3- phosphatase activity, sugar-phosphatase activity 3'(2'),5'-bisphosphate nucleotidase activity, phosphoric ester hydrolase activity

glycerol biosynthetic process, response to osmotic stress

sulfate assimilation, L-histidine biosynthetic process

Uroporphyrinogen decarboxylase hemE A0A0P0YK53 no N/A none predicted none predicted Farnesyltranstransferase A0AAN4R4W3 no N/A prenyltransferase activity isoprenoid biosynthetic process uncharacterized protein A0AAN4R4V8 no N/A metal ion binding none predicted

Glucokinase A0AAN4U1Z0 no N/A hexokinase activity none predicted 50S ribosomal protein L20 rplT A0AAN4R5H6 no N/A structural constituent of ribosome, rRNA binding translation

Site-determining protein minD A0AAN4R2Z7 no N/A ATP hydrolysis activity negative regulation of cell division

isomerase activity, carbohydrate binding, catalytic

Aldose 1-epimerase A0AAN4U2E0 no N/A activity carbohydrate metabolic process

Uracil phosphoribosyltransferase upp A0AAN4R645 no N/A uracil phosphoribosyltransferase activity uracil salvage

oxidoreductase activity, ferredoxin-NADP+

Ferredoxin--NADP reductase A0AAN4U235 no N/A reductase activity none predicted

Phosphoglycolate phosphatase pgp A0AAN4R2H9 no N/A phosphoglycolate phosphatase activity DNA repair, dephosphorylation 3-oxoacyl-ACP reductase A0AAN4R3D0 no N/A oxidoreductase activity none predicted

ferric uptake regulation protein fur A0A060QKA4 no N/A DNA-binding transcription factor activity regulation of DNA-templated transcription

phosphatidylinositol phosphate biosynthetic

Inositol-1-monophosphatase A0A060QE01 no N/A inositol monophosphate 1-phosphatase activity oxidoreductase activity, thioredoxin-disulfide

process

Thioredoxin reductase A0AAN4R4F8 no N/A reductase (NADPH) activity removal of superoxide radicals

NAD(P)-binding domain-containing protein A0A060QJ50 no N/A protein-containing complex binding ubiquinone predicted-6 biosynthetic process

Aldo/keto reductase A0AAN4R1X0 no N/A oxidoreductase activity none predicted Methionine aminopeptidase map A0A060QID3 no N/A metalloaminopeptidase activity proteolysis

oxidoreductase activity, flavin adenine

dinucleotide binding, oxidoreductase activity, acting on a sulfur group of donors, NAD(P) as

Glutathione-disulfide reductase gor A0AAN4U2D5 no N/A

Beta-ketoacyl-ACP reductase A0AAN4R2L2 no N/A Anaphase-promoting complex subunit 4-like

acceptor cell redox homeostasis

oxidoreductase activity, acting on the CH-OH

group of donors, NAD or NADP as acceptor fatty acid biosynthetic process

WD40 domain-containing protein A0A060QJ87 no N/A protein binding none predicted

Supplemental Table 3. All proteins identified under both growth conditions and all secretion predictions Gene Ontology Terms Gene Ontology Terms

| Protein Name | Gene Name Accession Predicted Secretion System | | | BastionHub Prediction Results | Molecular Function | Biological Process |
| --- | --- | --- | --- | --- | --- | --- |
| Entericidin | A0AAN4U355 I, II, III, IV, VI | | | 0.892, 0.654, 0.991, 0.667, 0.680 | none predicted | none predicted |
| Outer membrane protein | A0AAN4U3I6 I, II, IV | | | 1, 0.85, 0.765 | calcium ion binding | none predicted |
| TonB-dependent outer membrane siderophore receptor | A0AAN4U233 I, II | | | 0.616, 0.944 | none predicted | none predicted |
| TonB-dependent receptor | A0AAN4R251 I, II | | | 0.562, 0.904 | none predicted | none predicted |
| TonB-dependent outer membrane colicin I receptor | A0A0P0YI97 I, II, VI | | | 0.544, 0.918, 0.511 | none predicted | none predicted |
| Cell envelope biogenesis protein OmpA | A0A060QKK6 I, III | | | 0.935, 0.991 | none predicted | none predicted |
| YncE family protein | A0A0P0YE35 I, II | | | 0.787, 0.805 | none predicted | none predicted |
| flagellin | A0AAN4R5R2 I, II | | | 0.529, 0.569 | structural molecule activity | none predicted |
| Flagellar hook-associated protein 1 | A0AAN4QZU3 I ,II | | | 0.53, 0.718 | structural molecule activity | bacterial-type flagellum assembly |
| Flagellar hook protein FlgE D2 domain-containing protein | A0AAN4R0D8 I, II, III, IV, VI | | | 0.734, 0.933, 0.55, 0.502, 0.611 | none predicted | none predicted |
| TonB-dependent receptor | btuB A0AAN4R6T1 I, II | | | 0.628, 0.874 | none predicted | none predicted |
| Flagellar hook protein FlgE | flgE A0AAN4R3W6 I, II | | | 45292 | none predicted | bacterial-type flagellum-dependent swarming motility |
| TonB-dependent outer membrane siderophore receptor |  | A0A0N7KVB0 | I, II | 0.635, 0.904 | none predicted | none predicted |
| Lipoprotein |  | A0AAN4U3B7 | I, II, III, IV, VI | 0.924, 0.832, 0.936, 0.573, 0.614 | none predicted | none predicted |
| TonB-dependent receptor |  | A0AAN4R0V8 | I, II | 0.707, 0.944 | none predicted | none predicted |
| TonB-dependent receptor |  | A0A0P0YHI2 | I, II | 0.641, 1 | none predicted | none predicted |
| hypothetical protein |  | A0A0P0YFS9 | I, II, III, IV, VI | 0.738, 0.692, 0.91, 0.737, 0.938 | none predicted | none predicted |
| TonB-dependent receptor-like beta-barrel domain-containing protein |  | A0AAN4R2F9 | I, II, VI | 0.663, 0.918, 0.565 | siderophore uptake transmembrane transporter activity | siderophore transmembrane transport |
| Outer membrane protein |  | A0AAN4R2L9 | I, II, IV | 1, 0.633, 0.733 | none predicted | none predicted |
| uncharacterized protein |  | A0AAN4R5K2 | I, II, III, IV, VI | 0.647, 0.79, 0.831, 0.836, 0.865 | none predicted | none predicted |
| Glycine zipper domain-containing protein |  | A0AAN4U2F3 | I, II, III, IV, VI | 0.803, 0.694, 0.9, 0.676, 0.833 | none predicted | none predicted |
| Lipoprotein |  | A0A060QD82 | I, II, III, VI | 0.997, 0.785, 0.665, 0.706 | none predicted | none predicted |
| Lipoprotein |  | A0AAN4R288 | II, III, IV, VI | 0.896, 0.821, 0.689, 0.753 | none predicted | none predicted |
| aldose 1-epimerase |  | A0A0P0YEH3 | II | 0.825 | none predicted | none predicted |
| Catalase | katA | A0AAN4U282 | II, VI | 0.683, 0.828 | catalase activity, heme binding | response to oxidative stress, hydrogen peroxide catabolic process |
| peptidase M61 |  | A0AAN4R1M7 | II, VI | 0.99, 0.851 | protein binding | none predicted |
| FAS1 domain-containing protein |  | A0AAN4R766 | II | 0.792 | none predicted | none predicted |
| TonB-dependent receptor |  | A0A0P0YEY9 | II, VI | 0.944, 0.539 | none predicted | none predicted |
| Protein TolB | tolB | A0AAN4R0H1 | II | 0.954 | none predicted | protein transport |
| Lipoprotein |  | A0A060QEU8 | II | 0.736 | none predicted | none predicted |
| Lipase |  | A0AAN4R2H1 | II, III | 0.992, 0.538 | triacylglycerol lipase activity | lipid catabolic process |
|  |  |  |  |  | phosphatidylinositol binding, actin | actin filament bundle assembly, actin cortical patch localization, regulation of |
| Ysc84 actin-binding domain-containing protein |  | A0AAN4R2R6 | II | 0.624 | filament binding | ruffle assembly |
| TonB-dependent siderophore receptor |  | A0A0P0YG72 | II | 0.848 | none predicted | none predicted |
| membrane protein |  | A0AAN4R312 | II, VI | 0.978, 0.535 | none predicted | none predicted |
| Outer membrane protein assembly factor BamB | bamB | A0AAN4R100 | II | 0.749 | protein binding | none predicted |
| Glucans biosynthesis protein G |  | A0A060QIL1 | II, VI | 0.573, 0.526 | catalytic activity, carbohydrate binding | carbohydrate biosynthetic process, carbohydrate metabolic process |
| uncharacterized protein |  | A0AAN4R2A1 | II, VI | 0.528, 0.803 | none predicted | none predicted |
| DUF2501 domain-containing protein |  | A0A060QJA9 | II, III, VI | 0.786, 1, 0.79 | none predicted | none predicted |
| peptidoglycan lytic exotransglycosylase |  | A0AAN4U1L7 | II, VI | 0.654, 0.623 | hydrolase activity, hydrolyzing O- glycosyl compounds | peptidoglycan turnover |
| Probable outer membrane protein |  | A0A060QKA2 | II, VI | 0.92, 0.675 | none predicted | none predicted |
| Leucine-binding protein domain-containing protein |  | A0AAN4R4V6 | II | 0.738 | none predicted | none predicted |
| SH3 domain-containing protein |  | A0A0P0YJB3 | II, III, VI | 0.776, 0.944, 1 | none predicted | none predicted |
| DUF2125 domain-containing protein |  | A0AAN4R464 | II, III, IV, VI | 0.807, 1, 0.62, 0.846 | none predicted | none predicted |
| Phosphate-binding protein PstS | pstS | A0AAN4R2W7 | II | 0.971 | phosphate ion binding | phosphate ion transmembrane transport |
| Outer membrane protein OmpW |  | A0AAN4R498 | II | 0.86 | none predicted | none predicted |
| Gluconolaconase | xylC | A0AAN4R0F1 | II | 0.56 | gluconolactonase activity, calcium ion binding | L-ascorbic acid biosynthetic process |
| Porin |  | A0AAN4R2I7 | II, VI | 1, 0.571 | none predicted | none predicted |
| Ribonuclease I |  | A0AAN4R1H4 | II, VI | 0.856, 0.578 | RNA binding, ribonuclease T2 activity, RNA endonuclease activity | RNA catabolic process |
| 3-carboxymuconate cyclase |  | A0AAN4R2Y8 | II, III, VI | 0.971, 0.562, 0.555 | none predicted | protein binding |
| Lipoprotein |  | A0AAN4R1S0 | II, III, IV, VI | 0.836, 0.775, 0.676, 0.85 | none predicted | none predicted |
| uncharacterized protein |  | A0AAN4R2S7 | II | 0.548 | none predicted | none predicted |
| uncharacterized protein |  | A0AAN4R0W4 | II, III, IV, VI | 0.697, 0.976, 0.571, 0.936 | none predicted | none predicted |
| Peptidase |  | A0AAN4U369 | II | 0.65 | serine-type endopeptidase and peptidase activity | proteolysis |
| Gluconolaconase |  | A0AAN4R572 | II, VI | 0.773, 0.855 | none predicted | none predicted |
| lytic murein transglycosylase B |  | A0A0P0YG35 | II, VI | 0.886, 0.64 | none predicted | none predicted |
| uncharacterized protein |  | A0AAN4R021 | II | 0.632 | structural molecule activity | none predicted |
| TonB-dependent outer membrane siderophore receptor |  | A0AAN4R0C6 | II, VI | 0.944, 0.599 | none predicted | none predicted |
| C-type lysozyme inhibitor domain-containing protein |  | A0AAN4R1U7 | II, VI | 0.753, 0.534 | none predicted | none predicted |
|  |  |  |  |  | hydrolase activity, hydrolyzing O- glycosyl compounds, glycogen | carbohydrate metabolic process, |
| Glycogen debranching enzyme |  | A0A060QIY2 | II | 0.974 | debranching enzyme activity | glycogen catabolic process |
| uncharacterized protein |  | A0AAN4U3A6 | II, III, VI | 0.531, 0.826, 0.81 | none predicted | none predicted |
| DUF3465 domain-containing protein |  | A0AAN4R038 | II, III, VI | 0.769, 0.822, 1 | none predicted | none predicted |
| porin |  | A0AAN4U3E7 | II, VI | 1, 0.834 | porin activity | carbohydrate transport |
| uncharacterized protein |  | A0A060QCG9 | II III IV VI | 0.664, 1, 0.591, 0.847 | none predicted | none predicted |
| Secreted protein |  | A0AAN4R0G7 | II, III, IV, VI | 0.606, 0.999, 0.761, 0.935 | none predicted | none predicted |
| Endonuclease |  | A0AAN4R537 | II | 0.71 | nucleic acid binding, endonuclease activity, hydrolase activity, acting on ester bonds | DNA catabolic process |
| YfdX protein |  | A0AAN4R3K1 | II, III, IV, VI | 0.541, 1, 0.735, 0.683 | none predicted | none predicted |
| Maltose ABC transporter substrate-binding protein |  | A0AAN4R3M9 | II | 0.58 | none predicted | none predicted |
| Alginate biosynthesis protein AlgF |  | A0AAN4U3L3 | II, III, VI | 0.751, 0.653, 0.85 | none predicted | none predicted |
| Acid phosphatase |  | A0AAN4R1R1 | II, VI | 0.811, 0.761 | none predicted | none predicted |
| Ferric siderophore receptor | bfrH | A0AAN4R5L8 | II | 0.776 | siderophore-iron transmembrane transporter activity, signaling receptor activity | siderophore transport |
| Thioredoxin domain-containing protein |  | A0AAN4R1W8 | II, VI | 0.644, 0.598 | oxidoreductase activity | none predicted |
| pyrroloquinoline quinone predicted-dependent dehydrogenase |  | A0A0N7KVD1 | II, VI | 1, 0.924 | none predicted | none predicted |
| Polyisoprenoid-binding protein |  | A0AAN4R2U9 | II | 0.526 | none predicted | none predicted |
| Outer membrane protein assembly factor BamA | bamA | A0A060QGH4 | II | 0.555 | none predicted | membrane assembly, Gram-negative- bacterium-type cell outer membrane assembly |
| Glutaryl-7-ACA acylase | gaa | A0AAN4U2U5 | II, VI | 0.972, 0.548 | dipeptidyl-peptidase activity, hydrolase activity | none predicted |
| Levanase |  | A0AAN4R1X4 | II, VI | 1, 0.957 | hydrolase activity, hydrolyzing O- glycosyl compounds | carbohydrate metabolic process |
| Iron transport outer membrane receptor |  | A0AAN4R4Z7 | II | 0.817 | siderophore uptake transmembrane transporter activity | none predicted |
| Alpha,alpha-trehalase |  | A0AAN4U3F6 | II, VI | 0.96, 0.917 | alpha,alpha-trehalase activity | trehalose metabolic process, carbohydrate metabolic process |
| Gluconolactonase |  | A0AAN4R0S4 | II, VI | 0.821, 0.722 | none predicted | none predicted |
| uncharacterized protein |  | A0AAN4R4K7 | II, VI | 0.636 | none predicted | none predicted |
| uncharacterized protein |  | A0A060QCJ9 | III, IV | 0.967, 0.858 | none predicted | none predicted |
| MucR family transcriptional regulator |  | A0AAN4R381 | III | 0.773 | DNA binding, zinc ion binding | regulation of DNA-templated transcription |
| proteinase |  | A0AAN4R1W1 | III | 0.538 | metal ion binding | none predicted |
| uncharacterized protein |  | A0AAN4U2V8 | III, VI | 0.664, 0.616 | none predicted | none predicted |

| Outer membrane protein OmpH/Skp |  | A0AAN4U1G0 | III IV | 0.595, 0.515 | unfolded protein binding | none predicted |
| --- | --- | --- | --- | --- | --- | --- |
| uncharacterized protein |  | A0A060QM33 | III, VI | 1, 0.612 | none predicted | none predicted |
| Ankyrin repeat protein |  | A0AAN4R330 | III, IV, VI | 0.973, 0.845, 0.893 | none predicted | none predicted |
| Large ribosomal subunit protein uL29 | rpmC | A0A060QG13 | III | 0.684 | structural constituent of ribosomes | translation |
| Lipoprotein |  | A0AAN4R2L1 | III, IV, VI | 0.714, 0.743, 0.962 | none predicted | none predicted |
| DSBA oxidoreductase |  | A0AAN4R2A7 | III | 0.554 | oxidoreductase activity | none predicted |
| UrcA family protein |  | A0AAN4R0P5 | III, IV | 0.662, 0.716 | none predicted | none predicted |
| Periplasmic heavy metal sensor |  | A0AAN4R4L4 | III | 0.58 | unfolded protein binding | none predicted |
| uncharacterized protein |  | A0A060QDW4 | III, IV, VI | 0.975, 0.735, 0.54 | none predicted | none predicted |
| thiamine biosynthesis protein ThiC |  | A0A0P0YGH6 | III | 0.587 | none predicted | none predicted |
| Lipoprotein |  | A0AAN4R2Z3 | III | 0.535 | none predicted | none predicted |
| Integral membrane protein CcmA involved in cell shape determination |  | A0A060QLD3 | III | 0.51 | none predicted | none predicted |
| uncharacterized protein |  | A0A060QGF0 | III, IV | 0.948, 0.904 | none predicted | none predicted |
| DUF3126 domain-containing protein |  | A0A060QIP7 | III | 0.506 | none predicted | none predicted |
| DUF883 domain-containing protein |  | A0A060QK08 | III, IV | 0.983, 0.672 | none predicted | none predicted |
| DUF2934 domain-containing protein |  | A0A060QAY9 | III, IV, VI | 0.959, 0.941, 0.663 | none predicted | none predicted |
| Hypervirulence associated protein TUDOR domain-containing protein |  | A0A060QID8 | III, IV, VI | 0.559, 0.632, 0.632 | none predicted | none predicted |
| DUF1134 domain-containing protein |  | A0AAN4R5B0 | VI | 0.677 | none predicted | none predicted |
| Large ribosomal subunit protein bL27 | rpmA | A0A060QDR5 | VI | 0.621 | structural constituent of ribosome | translation |
| LysM/phospholipid-binding domain protein |  | A0A060QHZ6 | VI | 0.556 | none predicted | none predicted |
| Methionine adenosyltransferase |  | A0A433X0F8 | VI | 0.548 | methionine adenosyltransferase activity, ATP binding | S-adenosylmethionine biosynthet process |
| uncharacterized protein |  | A0AAN4R0E4 | VI | 0.567 | glycolipid transfer activity | lipopolysaccharide transport |
| Parvulin-like PPIase |  | A0A060QC01 | no | N/A | peptidyl-prolyl cis-trans isomerase activity | none predicted |
| uncharacterized protein |  | A0AAN4R1Q0 | no | N/A | none predicted | none predicted |
| Dipeptidyl carboxypeptidase II | dcp | A0AAN4U291 | no | N/A | peptidase activity, metalloendopeptidase activity | proteolysis |
| uncharacterized protein |  | A0AAN4U190 | no | N/A | none predicted | none predicted |
| Elongation factor Tu | tuf | A0A060QG23 | no | N/A | GTPase activity, GTP binding, translation elongation factor activity | translational elongation |
| Glyceraldehyde-3-phosphate dehydrogenase |  | A0A060QJP8 | no | N/A | NAD binding, oxidoreductase activity, acting on the aldehyde or oxo group of donors, NAD or NADP as acceptor | glucose metabolic process, |
| uncharacterized protein |  | A0AAN4R4B3 | no | N/A | none predicted | none predicted |
| Aconitate hydratase |  | A0AAN4R161 | no | N/A | none predicted | none predicted |
| uncharacterized protein |  | A0AAN4R3X0 | no | N/A | none predicted | none predicted |
| Chaperonin GroEL | groEL | A0A060QBJ4 | no | N/A | ATP-dependent protein folding chaperone | protein folding and refolding |
| Small ribosomal subunit protein uS19 | rpsS | A0A433WZ75 | no | N/A | structural constituent of ribosome | translation |
| transketolase |  | A0A060QI11 | no | N/A | transketolase activity | none predicted |
| N-ethylmaleimide reductase |  | A0A060QJK2 | no | N/A | FMN binding, oxidoreductase activity | none predicted |
| Peptidoglycan-associated lipoprotein | pal | A0A060QBC2 | no | N/A | none predicted | cell division |
| Aldehyde dehydrogenase |  | A0AAN4R3I6 | no | N/A | aldehyde dehydrogenase [NAD(P)+] activity, oxidoreductase activity | none predicted |
| Large ribosomal subunit protein uL11 | rplK | A0A060QHD3 | no | N/A | structural constituent of ribosome | translation |
| uncharacterized protein |  | A0AAN4R2T0 | no | N/A | none predicted | none predicted |
| Zinc metalloprotease |  | A0AAN4R6B2 | no | N/A | metalloendopeptidase activity, metallopeptidase activity | proteolysis |
| D-3-phosphoglycerate dehydrogenase |  | A0AAN4R4B4 | no | N/A | oxidoreductase activity, acting on the CH-OH group of donors, NAD or NADP as acceptor | none predicted |
| uncharacterized protein |  | A0AAN4R413 | no | N/A | none predicted | none predicted |
| Alkyl hydroperoxide reductase C |  | A0A060QKS4 | no | N/A | antioxidant activity, oxidoreductase activity, peroxiredoxin activity | response to oxidative stress |
| Non-specific DNA-binding protein Dps /  Iron-binding ferritin-like antioxidant protein / Ferroxidase |  | A0A060QKS1 | no | N/A | ferric iron binding, oxidoreductase activity, acting on metal ions | none predicted |
| Isocitrate dehydrogenase |  | A0AAN4U3I3 | no | N/A | magnesium ion binding, oxidoreductase activity, acting on the CH-OH group of donors, NAD or NADP as acceptor | none predicted |
| 6-phosphogluconate dehydrogenase,decarboxylating |  | A0A060QBX4 | no | N/A | NADP binding, phosphogluconate dehydrogenase (decarboxylating) activity | pentose-phosphate shunt |
| gamma-glutamyltranspeptidase |  | A0A0P0YI95 | no | N/A | none predicted | none predicted |
| Small ribosomal subunit protein uS12 | rpsL | A0A060QKQ0 | no | N/A | structural constituent of ribosome | translation |
| Large ribosomal subunit protein bL35 | rpmI | A0A060QGV0 | no | N/A | structural constituent of ribosome | translation |
| endopeptidase DegP/Do |  | A0A0P0YCQ8 | no | N/A | none predicted | none predicted |
| uncharacterized protein |  | A0AAN4R2G3 | no | N/A | none predicted | none predicted |
| Aminotransferase | hisC | A0AAN4R5F7 | no | N/A | pyridoxal phosphate binding | biosynthetic process |
| uncharacterized protein |  | A0AAN4R0X0 | no | N/A | none predicted | none predicted |
| Large ribosomal subunit protein bL28 | rpmB | A0A060QF16 | no | N/A | structural constituent of ribosome | translation |
| Arginine biosynthesis bifunctional protein ArgJ | argJ | A0AAN4U1Q8 | no | N/A | glutamate N-acetyltransferase activity | L-arginine biosynthetic process |
| Osmotically inducible protein C |  | A0A060QHA3 | no | N/A | peroxidase activity | response to oxidative stress |
| Chaperone protein DnaK | dnaK | A0AAN4R7N6 | no | N/A | ATP binding, unfolded protein binding, ATP-dependent protein folding chaperone | protein folding |
| Endoribonuclease L-PSP |  | A0A060QJF4 | no | N/A | none predicted | none predicted |
| Glucose-6-phosphate 1-dehydrogenase | zwf | A0AAN4U1I0 | no | N/A | oxidoreductase activity, acting on CH- OH group of donors, glucose-6- phosphate dehydrogenase activity, NADP binding | glucose metabolic process |
| Fructose-bisphosphate aldolase |  | A0AAN4R015 | no | N/A | lyase activity, fructose-bisphosphate aldolase activity | none predicted |
| OmpA-like domain-containing protein |  | A0A060QJL6 | no | N/A | none predicted | none predicted |
| Small ribosomal subunit protein bS20 | rpsT | A0A060QKE8 | no | N/A | structural constituent of ribosome | translation |
| Mannitol dehydrogenase | mtlD | A0AAN4U3M1 | no | N/A | oxidoreductase activity, catalytic activity | none predicted |
| NAD-dependent malic enzyme |  | A0A0P0YI47 | no | N/A | none predicted | none predicted |
| membrane integrity-associated transporter subunit PqiC |  | A0A0P0YDS7 | no | N/A | none predicted | none predicted |
| Large ribosomal subunit protein bL19 | rplS | A0A060QEQ5 | no | N/A | structural constituent of ribosome | translation |
| Superoxide dismutase |  | A0AAN4R0J4 | no | N/A | superoxide dismutase activity, metal ion binding | superoxide metabolic process |
| Elongation factor Ts | tsf | A0A060QK90 | no | N/A | translation elongation factor activity, protein binding | translational elongation |
| S-(Hydroxymethyl)glutathione dehydrogenase |  | A0AAN4U1Y7 | no | N/A | zinc ion binding, S-(hydroxymethyl) glutathione dehydrogenase [NAD(P)+] activity, oxidoreductase activity | formaldehyde catabolic process |
| Small ribosomal subunit protein uS17 | rpsQ | A0A060QHB2 | no | N/A | structural constituent of ribosome | translation |
| TPP-and FAD-dependent putative pyruvate dehydrogenase subunit A, |  | A0A0P0YI64 | no | N/A | none predicted | none predicted |
| Cold shock protein CspC |  | A0A060QJB1 | no | N/A | nucleic acid binding | none predicted |
| UTP--glucose-1-phosphate uridylyltransferase |  | A0AAN4U2V9 | no | N/A | UTP:glucose-1-phosphate uridylyltransferase activity | UDP-alpha-D-glucose metabolic process, biosynthetic process |
| Enolase | eno | A0AAN4R458 | no | N/A | magnesium ion binding, phosphopyruvate hydratase activity | glycolytic process |
| Large ribosomal subunit protein uL14 | rplN | A0A060QL79 | no | N/A | structural constituent of ribosome | translation |
| uncharacterized protein |  | A0AAN4U1E6 | no | N/A | protein binding | none predicted |
| Phosphoglucomutase, alpha-D-glucose phosphate-specific | celB | A0AAN4R0X4 | no | N/A | phosphoglucomutase activity, intramolecular phosphotransferase activity, magnesium ion binding | carbohydrate metabolic process |
| Large ribosomal subunit protein bL17 | rplQ | A0A433WZH2 | no | N/A | structural constituent of ribosome | translation |

ic

| Pyruvate kinase | pykF | A0AAN4R5E6 | no | N/A | magnesium ion binding, pyruvate kinase activity, potassium ion binding, catalytic  activity | glycolytic process |
| --- | --- | --- | --- | --- | --- | --- |
| Large ribosomal subunit protein bL33 | rpmG | A0A060QEQ7 | no | N/A | structural constituent of ribosome | translation |
| Peroxiredoxin |  | A0AAN4U159 | no | N/A | antioxidant activity, oxidoreductase activity | none predicted |
| Alcohol dehydrogenase |  | A0A060QEJ3 | no | N/A | oxidoreductase activity, acting on the CH-OH group of donors, NAD or NADP as acceptor, zinc ion binding | none predicted |
| Fructose-1,6-bisphosphatase |  | A0A060QK82 | no | N/A | fructose 1,6-bisphosphate 1- phosphatase activity | glycerol metabolic process, gluconeogenesis |
| Outer membrane protein assembly factor BamD | bamD | A0AAN4U381 | no | N/A | protein binding | none predicted |
| Large ribosomal subunit protein uL22 | rplV | A0A060QL81 | no | N/A | structural constituent of ribosome | translation |
| Glutamine synthetase |  | A0A060QLB9 | no | N/A | glutamine synthetase activity, catalytic activity | glutamine biosynthetic process |
| 50S ribosomal protein L18 | rplR | A0AAN4U2L5 | no | N/A | structural constituent of ribosome | translation |
| uncharacterized protein |  | A0AAN4R1S2 | no | N/A | peptidyl-prolyl cis-trans isomerase activity | none predicted |
| Outer-membrane lipoprotein carrier protein |  | A0A060QDZ8 | no | N/A | none predicted | none predicted |
| Phosphoglycerate kinase | pgk | A0AAN4R3L9 | no | N/A | phosphoglycerate kinase activity | glycolytic process |
| Transcriptional regulator, MarR family |  | A0A060QBI1 | no | N/A | DNA-binding transcription factor activity | regulation of DNA-templated transcription |
| Nucleoside diphosphate kinase | ndk | A0A060QBH3 | no | N/A | nucleoside diphosphate kinase activity | GTP biosynthetic process, UTP biosynthetic process, CTP biosynthetic process |
| uncharacterized protein |  | A0AAN4U1Y2 | no | N/A | none predicted | none predicted |
| Mannitol dehydrogenase | mtlD | A0AAN4R2Z0 | no | N/A | oxidoreductase activity, catalytic activity | mannitol metabolic process |
| 6-phosphogluconolactonase |  | A0AAN4U1E2 | no | N/A | 6-phosphogluconolactonase activity | carbohydrate metabolic process, pentose-phosphate shunt |
| uncharacterized protein |  | A0AAN4U1T5 | no | N/A | none predicted | none predicted |
| Small ribosomal subunit protein bS16 | rpsP | A0A060QE38 | no | N/A | structural constituent of ribosome | translation |
| Fumarate hydratase class II 2 | fumC2 | A0AAN4R470 | no | N/A | fumarate hydratase activity, lyase activity, catalytic activity | fumarate metabolic process, tricarboxylic acid cycle |
| Small ribosomal subunit protein bS18 | rpsR | A0A060QIA3 | no | N/A | structural constituent of ribosome | translation |
| Dipeptidyl-peptidase |  | A0AAN4U3J0 | no | N/A | dipeptidyl-peptidase activity, serine-type aminopeptidase activity | none predicted |
| ABC transporter ribose permease |  | A0A0P0YG43 | no | N/A | none predicted | none predicted |
| uncharacterized protein |  | A0AAN4U370 | no | N/A | none predicted | none predicted |
| Large ribosomal subunit protein uL6 | rplF | A0A060QL77 | no | N/A | structural constituent of ribosome | translation |
| Peptidase S41 |  | A0AAN4U2A6 | no | N/A | serine-type peptidase activity, protein binding | proteolysis |
| Small ribosomal subunit protein uS14 | rpsN | A0A060QG08 | no | N/A | structural constituent of ribosome | translation |
| glyoxalase/bleomycin resistance protein/dioxygenase |  | A0A0P0YE78 | no | N/A | none predicted | none predicted |
| Ribitol 2-dehydrogenase |  | A0A060QD11 | no | N/A | none predicted | none predicted |
| Small ribosomal subunit protein uS5 | rpsE | A0A060QGL7 | no | N/A | structural constituent of ribosome | translation |
| Large ribosomal subunit protein uL15 | rplO | A0A060QHA1 | no | N/A | structural constituent of ribosome | translation |
| uncharacterized protein |  | A0AAN4R5Z1 | no | N/A | peptidoglycan binding | none predicted |
| 2,3-bisphosphoglycerate-independent phosphoglycerate |  |  |  |  | phosphoglycerate mutase activity, manganese ion binding, catalytic |  |
| mutase | gpml | A0AAN4R5A9 | no | N/A | activity, metal ion binding | glucose catabolic process |
| Small ribosomal subunit protein uS10 | rpsJ | A0A060QHC3 | no | N/A | structural constituent of ribosome | translation |
| Nucleoside hydrolase |  | A0AAN4R136 | no | N/A | hydrolase activity, hydrolyzing N- glycosyl compounds | none predicted |
| Short-chain dehydrogenase |  | A0AAN4R3C5 | no | N/A | none predicted | none predicted |
| Oxidoreductase |  | A0AAN4U3L9 | no | N/A | oxidoreductase activity | none predicted |
| Serine hydroxymethyltransferase 2 | glyA 2 | A0AAN4R3R0 | no | N/A | glycine hydroxymethyltransferase activity, pyridoxal phosphate binding | glycine biosynthetic process from serine, tetrahydrofolate interconversion |
| uncharacterized protein |  | A0AAN4R1T3 | no | N/A | none predicted | lipoprotein transport |
| RND transporter |  | A0AAN4U3N7 | no | N/A | transmembrane transporter activity, efflux transmembrane transporter activity | transmembrane transport |
| Large ribosomal subunit protein uL24 | rplX | A0A433WZC1 | no | N/A | structural constituent of ribosome | translation |
| methylmalonate-semialdehyde dehydrogenase (CoA acylating) |  | A0A060QD80 | no | N/A | methylmalonate-semialdehyde dehydrogenase (acylating, NAD) activity, oxidoreductase acitivity, oxidoreductase activity, acting on the aldehyde or oxo group of donors, NAD or NADP as acceptor | none predicted |
| uncharacterized protein |  | A0AAN4R3S1 | no | N/A | none predicted | none predicted |
| branched-chain amino acid aminotransferase |  | A0A0N7KUJ6 | no | N/A | none predicted | none predicted |
| Ribose 5-phosphate isomerase B |  | A0A060QKL8 | no | N/A | isomerase activity | carbohydrate metabolic process |
| Methyltransferase |  | A0AAN4R2T7 | no | N/A | none predicted | none predicted |
| Large ribosomal subunit protein uL2 | rplB | A0A060QG18 | no | N/A | structural constituent of ribosome | translation |
| Bacterioferritin |  | A0A060QM49 | no | N/A | ferric iron binding | iron ion transport, intracellular iron ion homeostasis |
| Fructose-bisphosphate aldolase |  | A0A060QIK2 | no | N/A | zinc ion binding, aldehyde-lyase activity, fructose-bisphosphate aldolase activity | carbohydrate metabolic process, glycolytic process |
| Oxidoreductase |  | A0AAN4R2I8 | no | N/A | oxidoreductase activity | none predicted |
| UDP-N-acetylglucosamine 1-carboxyvinyltransferase | murA | A0AAN4R5D3 | no | N/A | UDP-N-acetylglucosamine 1- carboxyvinyltransferase activity, transferase activity, transferring alkyl or aryl (other than methyl) groups, catalytic activity | UDP-N-acetylgalactosamine biosynthetic process |
| Oxidoreductase |  | A0AAN4R134 | no | N/A | oxidoreductase activity | none predicted |
| Arylesterase |  | A0AAN4R2P4 | no | N/A | catalytic activity | none predicted |
| RND transporter MFP subunit |  | A0AAN4R2W3 | no | N/A | transmembrane transporter activity | transmembrane transport |
| Pyridine nucleotide-disulfide oxidoreductase |  | A0AAN4U2K0 | no | N/A | 2 iron, 2 sulfur cluster binding, oxidoreductase activity | none predicted |
| Trigger factor | tig | A0AAN4R4C8 | no | N/A | peptidyl-prolyl cis-trans isomerase activity | protein folding, protein transport |
| O-succinylhomoserine sulfhydrylase |  | A0A0P0YHT2 | no | N/A | none predicted | none predicted |
| uncharacterized protein |  | A0AAN4R119 | no | N/A | none predicted | none predicted |
| Large ribosomal subunit protein uL1 | rplA | A0A060QL87 | no | N/A | structural constituent of ribosome | translation |
| Histone-like DNA-binding protein |  | A0A060QMB0 | no | N/A | DNA binding, structural constituent of chromatin | none predicted |
| Thioredoxin | trxA | A0AAN4U2T7 | no | N/A | protein-disulfide reductase activity | none predicted |
| Small ribosomal subunit protein uS9 | rpsI | A0A060QCF7 | no | N/A | structural constituent of ribosome | translation |
| Glucose 1-dehydrogenase |  | A0A060QH73 | no | N/A | oxidoreductase activity | none predicted |
| Adenosine kinase |  | A0AAN4R1Q4 | no | N/A | kinase activity | none predicted |
| 3-isopropylmalate dehydrogenase | leuB | A0AAN4R3T0 | no | N/A | 3-isopropylmalate dehydrogenase activity, magnesium ion binding, oxidoreductase activity, acting on the CH-OH group of donors, NAD or NADP as acceptor, NAD binding | L-leucine biosynthetic process |
| Co-chaperonin GroES | groES | A0A060QG87 | no | N/A | ATP binding, protein folding chaperone | protein folding |
| Oxidoreductase |  | A0AAN4R261 | no | N/A | zinc ion binding, oxidoreductase activity | none predicted |
| uncharacterized protein |  | A0AAN4R504 | no | N/A | none predicted | none predicted |
| Large ribosomal subunit protein uL23 | rplW | A0A060QGN7 | no | N/A | structural constituent of ribosome | translation |
| Ribose-5-phosphate isomerase A | rpiA | A0AAN4R140 | no | N/A | structural constituent of ribosome | translation |
| Iron transporter |  | A0AAN4R551 | no | N/A | iron ion binding, iron-sulfur cluster binding | iron-sulfur cluster assembly |
| Elongation factor G | fusA | A0A060QDU2 | no | N/A | GTPase activity, GTP binding, translation elongation factor activity | translational elongation |

| Carbonic anhydrase |  | A0AAN4R3D6 | no | N/A | carbonate dehydratase activity, zinc ion  binding | carbon utilization |
| --- | --- | --- | --- | --- | --- | --- |
| Large ribosomal subunit protein uL3 | rplC | A0A060QL83 | no | N/A | structural constituent of ribosome | translation |
| Large ribosomal subunit protein uL5 | rplE | A0A060QGM4 | no | N/A | structural constituent of ribosome | translation |
| NAD(P)-dependent oxidoreductase |  | A0AAN4R2F6 | no | N/A | oxidoreductase activity | none predicted |
| Glucose-1-phosphate thymidylyltransferase |  | A0AAN4R444 | no | N/A | glucose-1-phosphate thymidylyltransferase activity | biosynthetic process |
| Small ribosomal subunit protein bS21 | rpsU | A0A060QED0 | no | N/A | structural constituent of ribosome | translation |
| 30S ribosomal protein S13 | rpsM | A0AAN4R629 | no | N/A | structural constituent of ribosome | translation |
| Ribosome-recycling factor | frr | A0AAN4R1F0 | no | N/A | none predicted | translation |
| Ketol-acid reductoisomerase (NADP(+)) | ilvC | A0AAN4R241 | no | N/A | ketol-acid reductoisomerase activity, oxidoreductase activity, NADP binding | branched-chain amino acid biosynthetic process |
| 50S ribosomal protein L25 | rplY | A0AAN4U470 | no | N/A | structural constituent of ribosome | translation |
| uncharacterized protein |  | A0AAN4U1R5 | no | N/A | hydrolase activity | none predicted |
| Electron transfer flavoprotein subunit alpha | etfA | A0AAN4U3L0 | no | N/A | electron transfer activity, flavin adenine dinucleotide binding | none predicted |
| ATP synthase subunit beta | atpD | A0A060QGJ6 | no | N/A | ATP binding, ATP hydrolysis activity, proton-transporting ATP synthase activity, rotational mechanism | ATP metabolic process, proton transmembrane transport, proton motive force-driven ATP synthesis |
| Large ribosomal subunit protein uL16 | rplP | A0A433WZ37 | no | N/A | structural constituent of ribosome | translation |
| 2,3,4,5-tetrahydropyridine-2,6-dicarboxylate |  |  |  |  | 2,3,4,5-tetrahydropyridine-2,6- dicarboxylate N-succinyltransferase | lysine biosynthetic process via |
| N-succinyltransferase | dapD | A0A060QGG0 | no | N/A | activity | diaminopimelate |
| 50S ribosomal protein L10 | rplJ | A0AAN4R616 | no | N/A | structural constituent of ribosome | translation |
| uncharacterized protein |  | A0AAN4R5I3 | no | N/A | phosphoprotein phosphatase activity | none predicted |
| CarD-like transcriptional regulator |  | A0A060QFU4 | no | N/A | none predicted | rRNA transcription |
| Nitroreductase |  | A0AAN4R608 | no | N/A | oxidoreductase activity | none predicted |
| AtsE protein |  | A0A060QDR1 | no | N/A | none predicted | none predicted |
| Ubiquinol oxidase subunit 2 | cyoA-2 | A0AAN4U2F9 | no | N/A | cytochrome-c oxidase activity, copper ion binding, oxidoreductase activity, acting on diphenols and related substances as donors, oxygen as acceptor, cytochrome bo3 ubiquinol oxidase activity | electron transport chain |
| Small ribosomal subunit protein uS4 | rpsD | A0A060QGK9 | no | N/A | structural constituent of ribosome | translation |
| Glucokinase | glk | A0AAN4R618 | no | N/A | glucokinase activity, ATP binding, D- glucose binding | glycolytic process, glucose 6-phosphate metabolic process |
| Small ribosomal subunit protein uS7 | rpsG | A0A060QGP2 | no | N/A | structural constituent of ribosome | translation |
| Alkyl hydroperoxide reductase subunit F |  | A0AAN4R2R0 | no | N/A | oxidoreductase activity, flavin adenine dinucleotide binding, NAD binding, NADH-dependent peroxiredoxin activity | response to reactive oxygen species |
| Orotate phosphoribosyltransferase | pyrE | A0AAN4R0P4 | no | N/A | orotate phosphoribosyltransferase activity | pyrimidine nucleotide biosynthetic process |
| Small ribosomal subunit protein uS3 | rpsC | A0A060QKP6 | no | N/A | structural constituent of ribosome | translation |
| Large ribosomal subunit protein bL9 | rplI | A0A060QGW6 | no | N/A | structural constituent of ribosome | translation |
| aldo/keto reductase |  | A0A0P0YDI1 | no | N/A | none predicted | none predicted |
|  |  |  |  |  | RNA binding, nucleic acid binding, polyribonucleotide | RNA processing, mRNA catabolic |
| Polyribonucleotide nucleotidyltransferase | pnp | A0AAN4R3B1 | no | N/A | nucleotidyltransferase activity | process |
| 50S ribosomal protein L4 | rplD | A0AAN4U2L2 | no | N/A | structural constituent of ribosome | translation |
| Adenosylhomocysteinase | ahcY | A0AAN4U3J2 | no | N/A | adenosylhomocysteinase activity | S-adenosylmethionine cycle |
| 6,7-dimethyl-8-ribityllumazine synthase | ribH | A0A060QCI0 | no | N/A | structural constituent of ribosome | translation |
| uncharacterized protein |  | A0AAN4R4U2 | no | N/A | none predicted | none predicted |
| Small ribosomal subunit protein uS11 | rpsK | A0A060QFZ7 | no | N/A | structural constituent of ribosome | translation |
| Dehydrogenase |  | A0AAN4R3U1 | no | N/A | none predicted | none predicted |
| 4-hydroxy-tetrahydrodipicolinate synthase | dapA | A0AAN4R671 | no | N/A | lyase activity, 4-hydroxy- tetrahydrodipicolinate synthase activity | lysine biosynthetic process via diaminopimelate |
| Two-component response regulator |  | A0AAN4R4X7 | no | N/A | none predicted | phosphorelay signal transduction system |
|  |  |  |  |  | carboxyl- or carbamoyltransferase activity, amino acid binding, ornithine | amino acid metabolic process, ornithine |
| Ornithine carbamoyltransferase | argF | A0AAN4R2U3 | no | N/A | carbamoyltransferase activity | metabolic process |
| class A beta-lactamase |  | A0A0P0YGZ0 | no | N/A | none predicted | none predicted |
| Succinate-semialdehyde dehydrogenase |  | A0AAN4R0B7 | no | N/A | aldehyde dehydrogenase [NAD(P)+] activity, oxidoreductase activity, oxidoreductase activity, acting on the aldehyde or oxo group of donors, NAD or NADP as acceptor | none predicted |
| Protein-export protein SecB | secB | A0A060QC78 | no | N/A | unfolded protein binding | protein transport, protein tetramerization |
| Dihydrolipoyl dehydrogenase |  | A0AAN4R451 | no | N/A | oxidoreductase activity, dihydrolipoyl dehydrogenase activity, flavin adenine dinucleotide binding, oxidoreductase activity, acting on a sulfur group of donors, NAD(P) as acceptor | none predicted |
| Acyl carrier protein | acpP | A0A060QG03 | no | N/A | none predicted | fatty acid biosynthetic process |
| Adenylosuccinate synthetase | purA | A0A060QDG3 | no | N/A | nucleotide binding, adenylosuccinate synthase activity, GTP binding | purine nucleotide biosynthetic process |
| Ribokinase | rbsK | A0AAN4U4C7 | no | N/A | structural constituent of ribosome | translation |
| Gluconate 5-dehydrogenase |  | A0AAN4QZZ2 | no | N/A | oxidoreductase activity | none predicted |
| Secretion protein HlyD |  | A0AAN4R3X2 | no | N/A | transmembrane transporter activity | transmembrane transport |
| Small ribosomal subunit protein uS8 | rpsH | A0A060QHA7 | no | N/A | structural constituent of ribosome | translation |
| Large ribosomal subunit protein bL12 | rplL | A0A060QGP9 | no | N/A | structural constituent of ribosome | translation |
| Aminotransferase |  | A0A060QK27 | no | N/A | pyridoxal phosphate binding, transaminase activity, catalytic activity | biosynthetic process, amino acid metabolic process |
| 2-dehydro-3-deoxyphosphogluconate aldolase/ 4-hydroxy-2-oxoglutarate aldolase |  | A0A0P0YGS3 | no | N/A | none predicted | none predicted |
| Inorganic pyrophosphatase | ppa | A0AAN4R535 | no | N/A | magnesium ion binding, inorganic diphosphate phosphatase activity | phosphate-containing compound metabolic process |
| cold shock proein |  | A0A060QCT0 | no | N/A | nucleic acid binding | none predicted |
| uncharacterized protein |  | A0AAN4U3T1 | no | N/A | catalytic activity | none predicted |
| 2-hydroxy-3-oxopropionate reductase | garR | A0AAN4U2B7 | no | N/A | NADP binding, NAD binding, oxidoreductase activity | none predicted |
| Tryptophan synthase alpha chain | trpA | A0A060QJC2 | no | N/A | tryptophan synthase activity | tryptophan metabolic process |
| Dihydrolipoyl dehydrogenase |  | A0AAN4R1C6 | no | N/A | oxidoreductase activity, dihydrolipoyl dehydrogenase activity, flavin adenine dinucleotide binding, oxidoreductase activity, acting on a sulfur group of donors, NAD(P) as acceptor | none predicted |
| Farnesyl-diphosphate synthase |  | A0AAN4U2K2 | no | N/A | prenyltransferase activity | isoprenoid biosynthetic process |
| Putative ABC transporter, periplasmic sugar binding protein |  | A0A060QCK3 | no | N/A | carbohydrate binding | none predicted |
| Dihydrolipoyllysine-residue succinyltransferase component of 2-oxoglutarate  dehydrogenase complex | odhB | A0AAN4R2E8 | no | N/A | acyltransferase activity, dihydrolipoyllysine-residue succinyltransferase activity | tricarboxylic acid cycle |
| RND transporter |  | A0AAN4U2D0 | no | N/A | transmembrane transporter activity, efflux transmembrane transporter activity | transmembrane transport |
| acetate/propionate family kinase |  | A0A0P0YGF8 | no | N/A | none predicted | none predicted |
| AdeC/adeK/oprM family multidrug efflux complex outer |  |  |  |  | transmembrane transporter activity, efflux transmembrane transporter |  |
| membrane factor | cusC | A0AAN4U2Q1 | no | N/A | activity | transmembrane transport |
| Small ribosomal subunit protein uS15 | rpsO | A0A060QKD5 | no | N/A | structural constituent of ribosome | translation |
| ABC transporter substrate-binding protein |  | A0A0P0YFG6 | no | N/A | none predicted | none predicted |
| Ribonuclease PH | rph | A0AAN4R3P2 | no | N/A | tRNA binding, tRNA nucleotidyltransferase activity | tRNA processing |
| Serine protease |  | A0AAN4U477 | no | N/A | protein binding, serine-type endopeptidase activity | proteolysis |

| uncharacterized protein |  | A0AAN4U398 | no | N/A | none predicted | none predicted |
| --- | --- | --- | --- | --- | --- | --- |
| Small ribosomal subunit protein bS6 | rpsF | A0A060QCT5 | no | N/A | structural constituent of ribosome | translation |
| oxidoreductase/SDR, 3-oxoacyl-[acyl carrier protein] reductase |  | A0A0P0YFM9 | no | N/A | none predicted | none predicted |
|  |  |  |  |  | orotidine-5'-phosphate decarboxylase | de novo' pyrimidine nucleobase biosynthetic process, 'de novo' UMP |
| Orotidine 5'-phosphate decarboxylase | pyrF | A0AAN4R749 | no | N/A | activity | biosynthetic process |
| Protein GrpE | grpE | A0AAN4R3P3 | no | N/A | adenyl-nucleotide exchange factor activity, protein homodimerization activity, protein-folding chaperone binding | protein folding |
| uncharacterized protein |  | A0AAN4R0M9 | no | N/A | none predicted | none predicted |
| Beta-ketoacyl-ACP reductase |  | A0AAN4U2I6 | no | N/A | 3-oxoacyl-[acyl-carrier-protein] reductase (NADPH) activity, NAD binding, oxidoreductase activity | fatty acid biosynthetic process |
| dCTP deaminase | dcd | A0A060QDS5 | no | N/A | dCTP deaminase activity | dUTP biosynthetic process |
| phosphate acetyl/butaryl transferase |  | A0A0P0YG52 | no | N/A | none predicted | none predicted |
| Cysteine synthase A |  | A0AAN4R262 | no | N/A | none predicted | cysteine biosynthetic process from serine |
| amidohydrolase |  | A0A0P0YIA8 | no | N/A | none predicted | none predicted |
| lipoprotein |  | A0AAN4U1Y1 | no | N/A | none predicted | none predicted |
| D-2-hydroxyacid dehydrogenase |  | A0AAN4R453 | no | N/A | oxidoreductase activity, acting on the CH-OH group of donors, NAD or NADP as acceptor, NAD binding | none predicted |
| Inositol monophosphatase |  | A0AAN4R5J4 | no | N/A | none predicted | phosphatidylinositol phosphate biosynthetic process |
| Phosphoribosylformylglycinamidine synthase subunit PurQ | purQ | A0A060QGZ2 | no | N/A | phosphoribosylformylglycinamidine synthase activity | de novo' IMP biosynthetic process |
| uncharacterized protein |  | A0AAN4U2X0 | no | N/A | acyltransferase activity, transferring groups other than amino-acyl groups, 1- (5-phosphoribosyl)-5-[(5- phosphoribosylamino) methylideneamino]imidazole-4- carboxamide isomerase activity | L-histidine biosynthetic process |
| Citrate synthase |  | A0A060QBT6 | no | N/A | citrate (Si)-synthase activity, acyltransferase activity, acyl groups converted into alkyl on transfer | tricarboxylic acid cycle |
| Phosphoribosylformylglycinamidine synthase subunit PurL | purL | A0AAN4R4N2 | no | N/A | phosphoribosylformylglycinamidine synthase activity | de novo' IMP biosynthetic process |
| Aminopeptidase |  | A0AAN4R1C3 | no | N/A | aminopeptidase activity | proteolysis |
| MBL fold hydrolase |  | A0AAN4U3W7 | no | N/A | none predicted | none predicted |
| Small ribosomal subunit protein uS2 | rpsB | A0A060QJ90 | no | N/A | structural constituent of ribosome | translation |
| Response regulator in two-component regulatory system |  |  |  |  |  | phosphorelay signal transduction system, regulation of DNA-templated |
| with PhoQ |  | A0A060QIV1 | no | N/A | DNA binding | transcription |
| Thioredoxin |  | A0AAN4R102 | no | N/A | protein binding | none predicted |
| Nitrogen regulatory protein P-II |  | A0A060QII8 | no | N/A | enzyme regulator activity | regulation of nitrogen utilization |
| Phosphoribosylamine--glycine ligase | purD | A0AAN4R362 | no | N/A | phosphoribosylamine-glycine ligase activity, ATP binding, metal ion binding | purine nucleobase biosynthetic process |
| uncharacterized protein |  | A0AAN4U2U4 | no | N/A | oxidoreductase activity | none predicted |
| Pyridoxine/pyridoxamine 5'-phosphate oxidase | pdxH | A0AAN4U1L8 | no | N/A | pyridoxamine phosphate oxidase activity, FMN binding, oxidoreductase activity, acting on the CH-NH2 group of donors | pyridoxine biosynthetic process |
| Lactoylglutathione lyase |  | A0A060QBR9 | no | N/A | lactoylglutathione lyase activity, metal ion binding | none predicted |
|  |  |  |  |  | GMP synthase (glutamine-hydrolyzing) activity, ATP binding, GMP synthase | GMP biosynthetic process, purine |
| GMP synthase [glutamine-hydrolyzing] | guaA | A0A060QC18 | no | N/A | activity | nucleotide biosynthetic process |
| Alcohol dehydrogenase | dhaT | A0AAN4U3E3 | no | N/A | oxidoreductase activity, metal ion binding | none predicted |
| Histidinol dehydrogenase | hisD | A0AAN4U3N9 | no | N/A | oxidoreductase activity, acting on the CH-OH group of donors, NAD or NADP as acceptor, metal ion binding, NAD binding, histidinol dehydrogenase activity, oxidoreductase activity | L-histidine biosynthetic process |
| Translation initiation factor IF-2 | infB | A0AAN4U1D2 | no | N/A | GTPase activity, GTP binding, translation initiation factor activity | translational initiation |
| DNA-binding protein HU-beta |  | A0A060QJ81 | no | N/A | DNA binding, structural constituent of chromatin | none predicted |
| uncharacterized protein |  | A0AAN4R454 | no | N/A | none predicted | none predicted |
| unknown function DUF461 domain protein |  | A0A0P0YFU8 | no | N/A | none predicted | none predicted |
| Cytosol aminopeptidase | pepA | A0AAN4R3L5 | no | N/A | metalloaminopeptidase activity, manganese ion binding, metal ion binding | proteolysis, protein metabolic process |
| NADH-ubiquinone predicted oxidoreductase chain E |  | A0A060QJS7 | no | N/A | oxidoreductase activity | none predicted |
| Amidophosphoribosyltransferase | purF | A0AAN4U3Q0 | no | N/A | amidophosphoribosyltransferase activity | purine nucleobase biosynthetic process |
| GTP-binding protein |  | A0AAN4R246 | no | N/A | GTP binding, GTPase activity | none predicted |
| 50S ribosomal protein L30 | rpmD | A0AAN4R2I0 | no | N/A | structural constituent of ribosome | translation |
| dTDP-glucose 4,6-dehydratase |  | A0A0P0YDI0 | no | N/A | none predicted | none predicted |

Supplemental Table 4. Gene Ontology (GO) terms associated with proteins on chocolate agar

Biological Process

No. of proteins with GO term

D-amino acid metabolic process 1

carbohydrate transport 3

glycerol metabolic process 1

none predicted 21

nucleoside triphosphate metabolic process 1

peptidoglycan metabolic process 1

protein import 1

proteolysis 3

No. of proteins with

Molecular Function GO term

D-amino-acid oxidase activity 1

FAD binding 1

acid phosphatase activity 1

catalytic activity 1

electron transfer activity and heme binding 1

flavin adenine dinucleotide binding 1

glycerone kinase activity and transferase activity 1

hydrolyzing O-glycosyl compounds 1

lytic transglycosylase activity and hydrolase activity 1

metallopeptidase activity 1

none predicted 16

nucleotide binding 1

oxidoreductase activity 1

porin activity 3

protein binding 1

protein binding and serine-type endopeptidase activity 1

ribonucleoside triphosphate phosphatase activity and nucleoside triphosphate diphosphatase 1

serine-type carboxypeptidase activity 2

tRNA binding 1

transferase activity 1

transferring phosphorus-containing groups 1

zinc ion binding 1

Supplemental Table 5. Gene Ontology (GO) terms associated with proteins on minimal media agar

Biological Process

No. of proteins with GO term

DNA binding 1

DNA catabolic process 1

DNA repair 1

L-histidine biosynthetic process 1

L-methionine salvage from methylthioadenosine 3

L-serine biosynthetic process 1

Mo-molybdopterin cofactor biosynthetic process 1

adenine salvage 1

bacterial-type flagellum-dependent cell motility 4

branched-chain amino acid biosynthetic process 1

carbohydrate metabolic process 5

cell division 1

cell redox homeostasis 1

cellular modified amino acid biosynthetic process 1

cellular response to oxidative stress 1

dTDP-rhamnose biosynthetic process 1

de novo' IMP biosynthetic process 1

dephosphorylation 1

extracellular polysaccharide biosynthetic process 1

flavin adenine dinucleotide binding 1

fatty acid biosynthetic process 2

glucan catabolic process 1

glutathione biosynthetic process 1

glycerol biosynthetic process 1

glycine biosynthetic process 1

isoprenoid biosynthetic process 1

negative regulation of cell division 1

none predicted 38

nucleobase-containing compound metabolic process 1

nucleoside metabolic process 1

oxidoreductase activity, acting on sulfur group of donors, NAD(P) as acceptor 1

oxidoreductase activity, acting on CH-OH group of donors, NAD or NADP as acceptor

pentose catabolic process 1

pentose-phosphate shunt 1

phosphatidylinositol phosphate biosynthetic process 2

polysaccharide biosynthetic process 1

protein folding 1

protein peptidyl-prolyl isomerization 1

protein repair 1

proteolysis 1

regulation of DNA-templated transcription 1

removal of superoxide radicals 1

response to osmotic stress 1

response to oxidative stress 1

structural constituent of chromatin 1

sulfate assimilation 1

sulfur compound metabolic process 1

translation 2

trehalose biosynthetic process 1

ubiquinone predicted-6 biosynthetic process 1

uracil salvage 1

xylulose metabolic process 1

No. of proteins

Molecular Function with GO term

3-oxoacyl-[acyl-carrier-protein] synthase activity 1

3'(2'),5'-bisphosphate nucleotidase activity 2

ATP binding 2

ATP hydrolysis activity 2

beta-glucosidase activity 1

D-ribulose-phosphate 3-epimerase activity 1

D-xylulokinase activity 1

DNA-binding transcription factor activity 1

DNA recombination 1

FMN binding 1

GTP binding 1

GTPase activity 1

L-methionine-(R)-S-oxide reductase activity 1

O-phospho-L-serine:2-oxoglutarate aminotransferase activity 1

S-methyl-5-thioadenosine phosphorylase activity 1

acireductone synthase activity 1

acting on NAD(P)H 1

acting on a sulfur group of donors 1

acting on carbohydrates and derivatives 1

acyltransferase activity, transferring groups other than amino-acyl groups 1

adenine phosphoribosyltransferase activity 1

adenylate kinase activity 1

aldehyde-lyase activity 2

aminopeptidase activity 1

carbohydrate binding 1

catalytic activity 4

cytoskeletal motor activity 1

dTDP-4-dehydrorhamnose 3,5-epimerase activity 1

disulfide as acceptor 1

disulfide oxidoreductase activity 2

electron transfer activity 1

exodeoxyribonuclease VII activity 1

ferredoxin-NADP+ reductase activity 1

glutamate-cysteine ligase activity 1

glycerol-1-phosphatase activity 1

glycerol-3-phosphatase activity 1

hexokinase activity 1

hydro-lyase activity 2

hydrolase activity 2

hydrolyzing O-glycosyl compounds 1

inositol monophosphate 1-phosphatase activity 1

isomerase activity 1

kinase activity 1

magnesium ion binding 2

metal ion binding 2

metalloaminopeptidase activity 1

none predicted 22

nucleobase-containing compound kinase activity 1

oxidoreductase activity 10

peptide-methionine (R)-S-oxide reductase activity 1

peptidoglycan binding 1

peptidyl-prolyl cis-trans isomerase activity 1

phosphatase activity 2

phosphate group as acceptor 1

phosphoglycolate phosphatase activity 1

phosphoribosylformylglycinamidine cyclo-ligase activity 1

phosphoric ester hydrolase activity 1

phosphotransferase activity 2

prenyltransferase activity 1

protein binding 2

protein-containing complex binding 1

quinone predicted or similar compound as acceptor 1

rRNA binding 1

racemase and epimerase activity 1

regulation of DNA-templated transcription 1

structural constituent of ribosome 2

structural molecule activity 1

sugar-phosphatase activity 1

thioredoxin-disulfide reductase (NADPH) activity 1

trehalose-phosphatase activity 1

uracil phosphoribosyltransferase activity 1

xanthine phosphoribosyltransferase activity 1

zinc ion binding 1

Supplemental Table 6. Gene Ontology (GO) terms associated with proteins on both conditions

Biological Process

No. of proteins with GO term

de novo' UMP biosynthetic process 1

ATP metabolic process 1

CTP biosynthetic process 1

DNA catabolic process 1

GMP biosynthetic process 1

GTP biosynthetic process 1

Gram-negative-bacterium-type cell outer membrane assembly 1

L-arginine biosynthetic process 1

L-ascorbic acid biosynthetic process 1

L-histidine biosynthetic process 2

L-leucine biosynthetic process 1

RNA catabolic process 1

RNA processing 1

S-adenosylmethionine biosynthetic process 1

S-adenosylmethionine cycle 1

UDP-N-acetylgalactosamine biosynthetic process 1

UDP-alpha-D-glucose metabolic process 1

UTP biosynthetic process 1

actin cortical patch localization 1

actin filament bundle assembly 1

amino acid metabolic process 3

bacterial-type flagellum assembly 1

bacterial-type flagellum-dependent swarming motility 1

biosynthetic process 4

branched-chain amino acid biosynthetic process 1

carbohydrate biosynthetic process 1

carbohydrate metabolic process 8

carbohydrate transport 1

carbon utilization 1

cell division 1

cell redox homeostasis 1

cysteine biosynthetic process from serine 1

dUTP biosynthetic process 1

de novo' IMP biosynthetic process 2

de novo' pyrimidine nucleobase biosynthetic process 1

electron transport chain 1

fatty acid biosynthetic process 3

formaldehyde catabolic process 1

fumarate metabolic process 1

gluconeogenesis 1

glucose 6-phosphate metabolic process 1

glucose catabolic process 1

glucose metabolic process 2

glutamine biosynthetic process 1

glycerol metabolic process 1

glycine biosynthetic process from serine 1

glycogen catabolic process 1

glycolytic process 5

hydrogen peroxide catabolic process 1

intracellular iron ion homeostasis 1

iron ion transport 1

iron-sulfur cluster assembly 2

isoprenoid biosynthetic process 1

lipid catabolic process 1

lipopolysaccharide transport 1

lipoprotein transport 1

lysine biosynthetic process via diaminopimelate 2

mRNA catabolic process 1

mannitol metabolic process 1

membrane assembly 1

none predicted 180

ornithine metabolic process 1

pentose-phosphate shunt 2

peptidoglycan turnover 1

phosphate ion transmembrane transport 1

phosphate-containing compound metabolic process 1

phosphatidylinositol phosphate biosynthetic process 1

phosphorelay signal transduction system 3

protein binding 1

protein folding 4

protein folding and refolding 1

protein metabolic process 1

protein tetramerization 1

protein transport 3

proteolysis 9

proton motive force-driven ATP synthesis 1

proton transmembrane transport 1

purine nucleobase biosynthetic process 2

purine nucleotide biosynthetic process 2

pyridoxine biosynthetic process 1

pyrimidine nucleotide biosynthetic process 1

rRNA transcription 1

regulation of DNA-templated transcription 3

regulation of nitrogen utilization 1

regulation of ruffle assembly 1

response to oxidative stress 3

response to reactive oxygen species 1

siderophore transmembrane transport 1

siderophore transport 1

superoxide metabolic process 1

tRNA processing 1

tetrahydrofolate interconversion 1

translation 50

translational elongation 3

translational initiation 1

transmembrane transport 5

trehalose metabolic process 1

tricarboxylic acid cycle 3

tryptophan metabolic process 1

No. of proteins

Molecular Function

1-(5-phosphoribosyl)-5-[(5-phosphoribosylamino)methylideneamino]imidazole-4-carboxamide

with GO Term

isomerase activity 1

2,3,4,5-tetrahydropyridine-2,6-dicarboxylate N-succinyltransferase activity 1

2 iron, 2 sulfur cluster binding 1

3-isopropylmalate dehydrogenase activity 1

3-oxoacyl-[acyl-carrier-protein] reductase (NADPH) activity 1

4-hydroxy-tetrahydrodipicolinate synthase activity 1

fructose 1,6-bisphosphate 1-phosphatase activity 1

6-phosphogluconolactonase activity 1

ATP binding 7

ATP hydrolysis activity 1

ATP-dependent protein folding chaperone 2

D-glucose binding 1

DNA binding 4

DNA-binding transcription factor activity 1

FMN binding 2

GMP synthase (glutamine-hydrolyzing) activity 1

GMP synthase activity 1

GTP binding 5

GTPase activity 4

NAD binding 7

NAD or NADP as acceptor 11

NAD(P) as acceptor 3

NADH-dependent peroxiredoxin activity 1

NADP binding 4

RNA binding 2

RNA endonuclease activity 1

S-(hydroxymethyl)glutathione dehydrogenase [NAD(P)+] activity 1

UDP-N-acetylglucosamine 1-carboxyvinyltransferase activity 1

UTP:glucose-1-phosphate uridylyltransferase activity 1

actin filament binding 1

acting on CH-OH group of donors 1

acting on a sulfur group of donors 3

acting on diphenols and related substances as donors 1

acting on ester bonds 1

acting on metal ions 1

acting on the CH-NH2 group of donors 1

acting on the CH-OH group of donors 7

acting on the aldehyde or oxo group of donors 3

acyl groups converted into alkyl on transfer 1

acyltransferase activity 3

adenosylhomocysteinase activity 1

adenyl-nucleotide exchange factor activity 1

| adenylosuccinate synthase activity | 1 |
| --- | --- |
| aldehyde dehydrogenase [NAD(P)+] activity | 2 |
| aldehyde-lyase activity | 1 |
| alpha,alpha-trehalase activity | 1 |
| amidophosphoribosyltransferase activity | 1 |
| amino acid binding | 1 |
| aminopeptidase activity | 1 |
| antioxidant activity | 2 |
| calcium ion binding | 2 |
| carbohydrate binding | 2 |
| carbonate dehydratase activity | 1 |
| carboxyl- or carbamoyltransferase activity | 1 |
| catalase activity | 1 |
| catalytic activity | 11 |
| citrate (Si)-synthase activity | 1 |
| copper ion binding | 1 |
| cytochrome bo3 ubiquinol oxidase activity | 1 |
| cytochrome-c oxidase activity | 1 |
| dCTP deaminase activity | 1 |
| dihydrolipoyl dehydrogenase activity | 2 |
| dihydrolipoyllysine-residue succinyltransferase activity | 1 |
| dipeptidyl-peptidase activity | 2 |
| efflux transmembrane transporter activity | 3 |
| electron transfer activity | 1 |
| endonuclease activity | 1 |
| enzyme regulator activity | 1 |
| ferric iron binding | 2 |
| flavin adenine dinucleotide binding | 5 |
| fructose-bisphosphate aldolase activity | 2 |
| fumarate hydratase activity | 1 |
| glucokinase activity | 1 |
| gluconolactonase activity | 1 |
| glucose-1-phosphate thymidylyltransferase activity | 1 |
| glucose-6-phosphate dehydrogenase activity | 1 |
| glutamate N-acetyltransferase activity | 1 |
| glutamine synthetase activity | 1 |
| glycine hydroxymethyltransferase activity | 1 |
| glycogen debranching enzyme activity | 1 |
| glycolipid transfer activity | 1 |
| heme binding | 1 |
| histidinol dehydrogenase activity | 1 |
| hydrolase activity | 7 |
| hydrolyzing N-glycosyl compounds | 1 |
| hydrolyzing O-glycosyl compounds | 3 |
| inorganic diphosphate phosphatase activity | 1 |
| intramolecular phosphotransferase activity | 1 |
| iron ion binding | 1 |
| iron-sulfur cluster binding | 1 |
| isomerase activity | 1 |

ketol-acid reductoisomerase activity 1

kinase activity 1

lactoylglutathione lyase activity 1

lyase activity 3

magnesium ion binding 6

manganese ion binding 2

metal ion binding 8

metalloaminopeptidase activity 2

metalloendopeptidase activity 2

metallopeptidase activity 1

methionine adenosyltransferase activity 1

methylmalonate-semialdehyde dehydrogenase (acylating, NAD) activity 1

none predicted 128

nucleic acid binding 4

nucleoside diphosphate kinase activity 1

nucleotide binding 1

ornithine carbamoyltransferase activity 1

orotate phosphoribosyltransferase activity 1

orotidine-5'-phosphate decarboxylase activity 1

oxidoreductase activity 49

oxygen as acceptor 1

peptidase activity 1

peptidoglycan binding 1

peptidyl-prolyl cis-trans isomerase activity 3

peroxidase activity 1

peroxiredoxin activity 1

phosphate ion binding 1

phosphatidylinositol binding 1

phosphoglucomutase activity 1

phosphogluconate dehydrogenase (decarboxylating) activity 1

phosphoglycerate kinase activity 1

phosphoglycerate mutase activity 1

phosphoprotein phosphatase activity 1

phosphopyruvate hydratase activity 1

phosphoribosylamine-glycine ligase activity 1

phosphoribosylformylglycinamidine synthase activity 2

polyribonucleotide nucleotidyltransferase activity 1

porin activity 1

potassium ion binding 1

prenyltransferase activity 1

protein binding 8

protein folding chaperone 1

protein homodimerization activity 1

protein-disulfide reductase activity 1

protein-folding chaperone binding 1

proton-transporting ATP synthase activity 1

pyridoxal phosphate binding 4

pyridoxamine phosphate oxidase activity 1

pyruvate kinase activity 1

| ribonuclease T2 activity | 1 |
| --- | --- |
| rotational mechanism | 1 |
| serine-type aminopeptidase activity | 1 |
| serine-type carboxypeptidase activity | 1 |
| serine-type endopeptidase activity | 1 |
| serine-type endopeptidase and peptidase activity | 1 |
| serine-type peptidase activity | 1 |
| siderophore uptake transmembrane transporter activity | 2 |
| siderophore-iron transmembrane transporter activity | 1 |
| signaling receptor activity | 1 |
| single-stranded DNA binding | 1 |
| structural constituent of chromatin | 1 |
| structural constituent of ribosome | 48 |
| structural constituent of ribosomes | 1 |
| structural molecule activity | 3 |
| superoxide dismutase activity | 1 |
| tRNA binding | 1 |
| tRNA nucleotidyltransferase activity | 1 |
| transaminase activity | 2 |
| transferase activity | 1 |
| transferring alkyl or aryl (other than methyl) groups | 1 |
| transferring groups other than amino-acyl groups | 1 |
| transketolase activity | 1 |
| translation elongation factor activity | 3 |
| translation initiation factor activity | 1 |
| transmembrane transporter activity | 5 |
| triacylglycerol lipase activity | 1 |
| tryptophan synthase activity | 1 |
| ubiquinone-6 biosynthetic process | 1 |
| unfolded protein binding | 4 |
| zinc ion binding | 6 |

Supplementary Table 7. Proteins identified in only a single biological replicate and prediction of secretion

Predicted Secretion BastionHub

| Growth media | Protein Name | Gene Name | Accession | System | Prediction Results |
| --- | --- | --- | --- | --- | --- |
| Chocolate agar | Peptidase |  | A0AAN4R2K1 | II | 0.843 |
| Chocolate agar | TonB-dependent receptor |  | A0AAN4R5C4 | II | 0.822 |
| Chocolate agar | Peptidase M23 domain-containing protein |  | A0AAN4R283 | II | 0.584 |
| Chocolate agar | Signal peptidase |  | A0AAN4R151 | III | 0.924 |
| Chocolate agar | Hypothetical protein |  | A0A0P0YDV3 | III | 1 |
| Chocolate agar | Dihydroxyacetone kinase |  | A0AAN4R2Z2 | no | N/A |
| Chocolate agar | 30S ribosomal protein S1 | rpsA | A0AAN4R0D9 | no | N/A |
| Chocolate agar | Aspartyl/glutamyl-tRNA(Asn/Gln) amidotransferase subunit B | gatB | A0AAN4R651 | no | N/A |
| Chocolate agar | Aldehyde dehydrogenase | aldA | A0AAN4U198 | no | N/A |
| Chocolate agar | Sugar phosphate isomerases/epimerase IolH | iolH | A0AAN4U3P4 | no | N/A |
| Chocolate agar | Inosine-5'-monophosphate dehydrogenase | guaB | A0AAN4R639 | no | N/A |
| Chocolate agar | Threonine--tRNA ligase | thrS | A0AAN4U3T4 | no | N/A |
| Chocolate agar | Adenylosuccinate lyase | purB | A0AAN4U384 | no | N/A |
| Chocolate agar | Adenylyl-sulfate kinase | nodQ | A0AAN4U188 | no | N/A |
| Chocolate agar | Carbamoyl-phosphate synthase (Glutamine-hydrolyzing) | carB | A0AAN4U1B9 | no | N/A |
| Chocolate agar | Ribonuclease E | rne | A0A060QGA6 | no | N/A |
| Chocolate agar | Molybdenum cofactor biosynthesis protein |  | A0AAN4R1G5 | no | N/A |
| Chocolate agar | Glutamate--tRNA ligase | gltX2 | A0AAN4R3I4 | no | N/A |
| Chocolate agar | ATP-dependent Clp protease proteolytic subunit | clpP | A0AAN4R682 | no | N/A |
| Chocolate agar | Lysine--tRNA ligase | lysS | A0AAN4R4Q9 | no | N/A |
| Chocolate agar | Sorbitol 6-phosphate dehydrogenase |  | A0AAN4R0Y3 | no | N/A |
| Chocolate agar | 2-oxoglutarate dehydrogenase subunit E1 | sucA | A0AAN4R2F4 | no | N/A |
| Chocolate agar | Large ribosomal subunit protein bL32 | rpmF | A0A060QF09 | no | N/A |
| Chocolate agar | Pyruvate dehydrogenase E1 component subunit alpha | pdhA | A0AAN4R439 | no | N/A |
| Chocolate agar | Uncharacterized protein |  | A0AAN4R3U4 | no | N/A |
| Chocolate agar | Pyruvate dehydrogenase complex E1 component subunit beta |  | A0AAN4R6V8 | no | N/A |
| Chocolate agar | RND transporter |  | A0AAN4R582 | no | N/A |
| Chocolate agar | Isoleucine--tRNA ligase | ileS | A0AAN4U2Q6 | no | N/A |
| Chocolate agar | Transcription termination factor Rho | rho | A0AAN4U3H9 | no | N/A |
| Chocolate agar | Peptide chain release factor 2 | prfB | A0AAN4U1S8 | no | N/A |
| Chocolate agar | Signal recognition particle protein | ffh | A0A060QDH2 | no | N/A |
| Chocolate agar | D-alanyl-D-alanine carboxypeptidase |  | A0AAN4R3Y6 | no | N/A |
| Chocolate agar | Glutamine--fructose-6-phosphate aminotransferase [isomerizing] | glmS | A0AAN4R5Z5 | no | N/A |
| Chocolate agar | Delta-aminolevulinic acid dehydratase |  | A0AAN4R4J5 | no | N/A |
| Chocolate agar | Acetylglutamate kinase | argB | A0AAN4R186 | no | N/A |
| Chocolate agar | peptidase M20 |  | A0AAN4R761 | no | N/A |
| Chocolate agar | Alcohol dehydrogenase |  | A0AAN4R4T7 | no | N/A |
| Chocolate agar | Trehalose-6-phosphate synthase |  | A0AAN4U3J6 | no | N/A |
| Chocolate agar | Carboxymethylenebutenolidase |  | A0AAN4R1J3 | no | N/A |
| Chocolate agar | Protein translocase subunit SecE | secE | A0A060QGK1 | no | N/A |
| Chocolate agar | MexX family efflux pump subunit |  | A0AAN4U2X4 | no | N/A |
| Chocolate agar | Gamma-glutamyl phosphate reductase | proA | A0AAN4R3V5 | no | N/A |
| Minimal agar | Gluconolactonase |  | A0AAN4R1L0 | II | 0.828 |
| Minimal agar | Superoxide dismutase [Cu-Zn] | sodC | A0AAN4R281 | II | 0.647 |
| Minimal agar | Aldose 1-epimerase |  | A0AAN4R3Y7 | II | 0.597 |
| Minimal agar | Uncharacterized protein |  | A0AAN4U3X4 | III | 0.897 |
| Minimal agar | NADPH-dependent oxidoreductase |  | A0AAN4U374 | no | N/A |
| Minimal agar | Glycerol-3-phosphatase |  | A0AAN4R4Q5 | no | N/A |
| Minimal agar | Transcription elongation factor GreA | greA | A0AAN4U1P3 | no | N/A |
| Minimal agar | ATP synthase subunit alpha | atpA | A0AAN4U1S9 | no | N/A |
| Minimal agar | Gluconokinase |  | A0AAN4R3J1 | no | N/A |
| Minimal agar | N-(5'-phosphoribosyl)anthranilate isomerase | trpF | A0AAN4R4S7 | no | N/A |
| Minimal agar | Response regulator |  | A0AAN4R005 | no | N/A |
| Minimal agar | Thiamine-phosphate synthase | thiE | A0AAN4U2Y8 | no | N/A |
| Minimal agar | 2-nitropropane dioxygenase |  | A0AAN4R2S6 | no | N/A |
| Minimal agar | Cell division topological specificity factor | minE | A0AAN4R606 | no | N/A |
| Minimal agar | Ferredoxin--NADP(+) reductase | fpr-2 | A0AAN4U2P6 | no | N/A |
| Minimal agar | uncharacterized protein |  | A0AAN4R2Y2 | no | N/A |
| Minimal agar | Peptide methionine sulfoxide reductase MsrA | msrA | A0AAN4U333 | no | N/A |
| Minimal agar | DNA-binding response regulator |  | A0AAN4R2W8 | no | N/A |
| Minimal agar | ABC transporter ATP-binding protein | sufC | A0AAN4U331 | no | N/A |
| Minimal agar | thiazole synthase | thiG | A0AAN4U2Z6 | no | N/A |
| Minimal agar | Aminotransferase V |  | A0AAN4U3K2 | no | N/A |
| Minimal agar | Dihydroorotase | pyrC | A0AAN4R2R8 | no | N/A |
| Minimal agar | 4-diphosphocytidyl-2-C-methyl-D-erythritol kinase | ispE | A0AAN4R1U5 | no | N/A |

| Minimal agar | Ubiquinone predicted-binding protein |  | A0AAN4R3G1 | no | N/A |
| --- | --- | --- | --- | --- | --- |
| Minimal agar | Inosine-uridine preferring nucleoside hydrolase |  | A0A060QCN2 | no | N/A |
| Minimal agar | ATP synthase subunit delta | atpH | A0AAN4U230 | no | N/A |
| Minimal agar | Carboxymethylenebutenolidase |  | A0AAN4U1L4 | no | N/A |
| Minimal agar | DNA-directed RNA polymerase subunit omega | rpoZ | A0A060QB82 | no | N/A |
| Minimal agar | UDP-N-acetylglucosamine--N-acetylmuramyl-(pentapeptide) pyrophosphoryl-undecaprenol | murG | A0AAN4R787 | no | N/A |
|  | N-acetylglucosamine transferase |  |  |  |  |
| Minimal agar | Xylose isomerase |  | A0AAN4U2Y1 | no | N/A |
| Minimal agar | Pyrroline-5-carboxylate reductase | proC | A0AAN4R510 | no | N/A |
| Minimal agar | 3-isopropylmalate dehydratase large subunit | leuC | A0AAN4R4K0 | no | N/A |
| Minimal agar | Integration host factor subunit alpha | ihfA | A0A060QJ49 | no | N/A |
| Minimal agar | 8-amino-7-oxononanoate synthase |  | A0AAN4U2M8 | no | N/A |
| Minimal agar | Deoxyuridine 5'-triphosphate nucleotidohydrolase | dut | A0AAN4U3U6 | no | N/A |
| Minimal agar | Xaa-Pro aminopeptidase |  | A0AAN4U3F0 | no | N/A |
| Minimal agar | Phosphoribosylformylglycinamidine synthase subunit PurS | purS | A0AAN4U323 | no | N/A |
| Minimal agar | 2-dehydro-3-deoxyphosphooctonate aldolase | kdsA | A0AAN4U2T9 | no | N/A |
| Minimal agar | N utilization substance protein B | nusB | A0AAN4R2R7 | no | N/A |
| Minimal agar | 2-nitropropane dioxygenase |  | A0AAN4R171 | no | N/A |
| Minimal agar | 4-hydroxy-tetrahydrodipicolinate reductase | dapB | A0AAN4R409 | no | N/A |
| Minimal agar | RNA-binding protein Hfq | hfq | A0A060QHV3 | no | N/A |
| Minimal agar | Aspartate--tRNA(Asp/Asn) ligase | aspS | A0AAN4U2P1 | no | N/A |
| Both Conditions | Purine nucleoside permease |  | A0AAN4QZZ8 | II | 0.925 |
| Both Conditions | Single-stranded DNA-binding protein |  | A0A060QI44 | II | 0.598 |
| Both Conditions | Phage protein |  | A0AAN4U327 | III | 0.771 |
| Both Conditions | Glutathione peroxidase |  | A0A060QBY1 | III | 0.932 |
| Both Conditions | Acyl carrier protein |  | A0A060QFG2 | III | 0.555 |
| Both Conditions | TIGR02300 family protein |  | A0AAN4R095 | no | N/A |
| Both Conditions | Arginine--tRNA ligase | argS | A0AAN4R1W5 | no | N/A |
| Both Conditions | Argininosuccinate synthase | argG | A0A060QFT0 | no | N/A |
| Both Conditions | Enoyl-[acyl-carrier-protein] reductase [NADH] | fabI | A0AAN4U1Q0 | no | N/A |
| Both Conditions | Chaperone protein ClpB | clpB | A0AAN4R416 | no | N/A |
| Both Conditions | Large ribosomal subunit protein uL13 | rplM | A0A060QD18 | no | N/A |
| Both Conditions | Diacetyl reductase | budC | A0AAN4U3K0 | no | N/A |
| Both Conditions | Energy-dependent translational throttle protein EttA | ettA | A0A060QKU8 | no | N/A |
| Both Conditions | uncharacterized protein |  | A0AAN4R3T9 | no | N/A |
| Both Conditions | Homoserine dehydrogenase |  | A0AAN4R534 | no | N/A |
| Both Conditions | Iron-sulfur cluster carrier protein |  | A0AAN4R529 | no | N/A |
| Both Conditions | DNA polymerase III subunit beta |  | A0AAN4R6X4 | no | N/A |
| Both Conditions | Protein-export protein secB 2 |  | A0A060QD79 | no | N/A |
| Both Conditions | NADPH-dependent 7-cyano-7-deazaguanine reductase | queF | A0A060QKW2 | no | N/A |
| Both Conditions | Exopolyphosphatase |  | A0AAN4U2G9 | no | N/A |
| Both Conditions | Bifunctional purine biosynthesis protein PurH | purH | A0AAN4U2Z2 | no | N/A |
| Both Conditions | Alpha-hydroxy-acid oxidizing enzyme | lldD | A0AAN4U1J5 | no | N/A |
| Both Conditions | uncharacterized protein |  | A0AAN4R379 | no | N/A |
| Both Conditions | Elongation factor P | efp | A0A060QIT0 | no | N/A |
| Both Conditions | DNA-binding response regulator |  | A0AAN4R221 | no | N/A |
| Both Conditions | Biotin carboxyl carrier protein of acetyl-CoA carboxylase |  | A0A060QMB7 | no | N/A |
| Both Conditions | Oxidoreductase |  | A0AAN4U2D3 | no | N/A |
| Both Conditions | Translation initiation factor IF-3 | infC | A0A433WS83 | no | N/A |
| Both Conditions | Glutamyl-tRNA(Gln) amidotransferase subunit A | gatA | A0AAN4R488 | no | N/A |
| Both Conditions | Aerobic cobaltochelatase CobS subunit |  | A0A060QJC1 | no | N/A |
| Both Conditions | D-tagatose-bisphosphate aldolase, class II, non-catalytic subunit |  | A0AAN4R3W0 | no | N/A |
| Both Conditions | NADH-quinone predicted oxidoreductase |  | A0AAN4R292 | no | N/A |
| Both Conditions | Phosphate-specific transport system accessory protein PhoU |  | A0A060QHH9 | no | N/A |
| Both Conditions | Protein RecA | recA | A0AAN4R1H5 | no | N/A |
| Both Conditions | 3-phosphoshikimate 1-carboxyvinyltransferase |  | A0A0P0YJ00 | no | N/A |
| Both Conditions | DNA-binding response regulator |  | A0AAN4U317 | no | N/A |
| Both Conditions | DNA-directed RNA polymerase subunit beta | rpoB | A0AAN4R1U1 | no | N/A |
| Both Conditions | 2-isopropylmalate synthase | leuA | A0AAN4U3E5 | no | N/A |
| Both Conditions | Threonylcarbamoyl-AMP synthase |  | A0A060QGF3 | no | N/A |
